# Evaluating the Acceptance and Usability of an Independent, Noncommercial Search Engine for Medical Information: Cross-Sectional Questionnaire Study and User Behavior Tracking Analysis

**DOI:** 10.2196/56941

**Published:** 2025-01-23

**Authors:** Lisa Specht, Raphael Scheible, Martin Boeker, Erik Farin-Glattacker, Nikolas Kampel, Marina Schmölz, Andrea Schöpf-Lazzarino, Stefan Schulz, Christian Schlett, Fabian Thomczyk, Sebastian Voigt-Radloff, Constanze Wegner, Katharina Wollmann, Andy Maun

**Affiliations:** 1 Institute of General Practice Faculty of Medicine and Medical Center University of Freiburg Freiburg Germany; 2 Institute of Artificial Intelligence and Informatics in Medicine Chair of Medical Informatics, University Hospital rechts der Isar, School of Medicine and Health Technical University of Munich Munich Germany; 3 Institute of Medical Biometry and Statistics, Section of Health Care Research and Rehabilitation Research Faculty of Medicine and Medical Center University of Freiburg Freiburg Germany; 4 Institute of Neuroscience and Medicine Jülich Aachen Research Alliance Forschungszentrum Jülich GmbH Jülich Germany; 5 Careum School of Health part of the Kalaidos University of Applied Sciences Zurich Switzerland; 6 Institute for Medical Informatics Statistics and Documentation Medical University of Graz Graz Austria; 7 Data Integration Center University of Freiburg Freiburg Germany; 8 Cochrane Germany Cochrane Germany Foundation Freiburg Germany

**Keywords:** medical information, health information, search engine, user behavior, health literacy, digital health literacy, navigational needs, information-seeking behavior, evidence-based content, Germany

## Abstract

**Background:**

The internet is a key source of health information, but the quality of content from popular search engines varies, posing challenges for users—especially those with low health or digital health literacy. To address this, the “tala-med” search engine was developed in 2020 to provide access to high-quality, evidence-based content. It prioritizes German health websites based on trustworthiness, recency, user-friendliness, and comprehensibility, offering category-based filters while ensuring privacy by avoiding data collection and advertisements.

**Objective:**

This study aims to evaluate the acceptance and usability of this independent, noncommercial search engine from the users’ perspectives and their actual use of the search engine.

**Methods:**

For the questionnaire study, a cross-sectional study design was used. In total, 802 participants were recruited through a web-based panel and were asked to interact with the new search engine before completing a web-based questionnaire. Descriptive statistics and multiple regression analyses were used to assess participants’ acceptance and usability ratings, as well as predictors of acceptance. Furthermore, from October 2020 to June 2021, we used the open-source web analytics platform Matomo to collect behavior-tracking data from consenting users of the search engine.

**Results:**

The study indicated positive findings on the acceptance and usability of the search engine, with more than half of the participants willing to reuse (465/802, 58%) and recommend it (507/802, 63.2%). Of the 802 users, 747 (93.1%) valued the absence of advertising. Furthermore, 92.3% (518/561), 93.9% (553/589), 94.7% (567/599), and 96.5% (600/622) of those users who used the filters agreed at least partially that the filter functions were helpful in finding trustworthy, recent, user-friendly, or comprehensible results. Participants criticized some of the search results regarding the selection of domains and shared ideas for potential improvements (eg, for a clearer design). Regression analyses showed that the search engine was especially well accepted among older users, frequent internet users, and those with lower educational levels, indicating an effective targeting of segments of the population with lower health literacy and digital health literacy. Tracking data analysis revealed 1631 sessions, comprising 3090 searches across 1984 unique terms. Users performed 1.64 (SD 1.31) searches per visit on average. They prioritized the search terms “corona,” “back pain,” and “cough.” Filter changes were common, especially for *recency* and *trustworthiness*, reflecting the importance that users placed on these criteria.

**Conclusions:**

User questionnaires and behavior tracking showed the platform was well received, particularly by older and less educated users, especially for its advertisement-free design and filtering system. While feedback highlighted areas for improvement in design and filter functionality, the search engine’s focus on transparency, evidence-based content, and user privacy shows promise in addressing health literacy and navigational needs. Future updates and research will further refine its effectiveness and impact on promoting access to quality health information.

## Introduction

### Background

The internet has become a crucial resource for accessing medical information, making it a valuable tool for individuals seeking knowledge about health. In Germany, medical professionals, notably physicians, have long been the primary source of health care information for the general population [1,2]. However, the landscape is evolving, and the internet now stands as the second most significant channel for health-related information [1], indicating a new trend in how people seek medical knowledge. A study conducted by the Bertelsmann Foundation showed a strong and increasing demand for health-related information online, including a rapidly increasing demand by older generations [3]. In 2020, approximately 70% of the German population used the internet to actively engage in searching for health-related content [4]. While these numbers have varied in recent years, there is an overall trend of an increased use of the internet [4].

A major complaint that users have about health-related information online is the lack of clarity regarding the trustworthiness and seriousness of existing websites. The information online has varying levels of quality [5,6]. Frequently used and popular websites are regarded as reliable by users, irrespective of the actual quality of their content, while independent public websites are not as well known and do not seem to be more reliable to users [3]. The reliance on digital platforms for health information also poses a challenge due to the profound influence of the order of the results generated by popular search engines. Users often initiate their search for medical information through well-known search engines, predominantly relying on the first few search results displayed on the search engine results page (SERP), which are often advertising [7-10]. Unfortunately, these top hits are not only clicked on more frequently but are also perceived as more trustworthy, despite the variation in information quality [3,9]. This highlights the ambiguity of users feeling incompetent in finding reliable information online, while at the same time trusting the top results and being satisfied with the information they find. A more recent study evaluated the quality of online health and nutrition information related to cancer supplements through a Google search, using the Health Information Quality Index [11]. The 160 relevant search results yielded median and mean Health Information Quality Index scores of 8, with one-quarter of the results scoring high (10-12). No correlation was found between high quality scores and an early appearance of these results, indicating potential limitations in using Google for obtaining accurate information on dietary supplements and cancer, particularly given the prevalence of advertisements outnumbering search results [11].

The problem with the reliance on search engines becomes even clearer when considering the low health literacy and digital health literacy of the German population [12-14]. The first Health Literacy Survey Germany study in 2014 [13] underscored a significant health literacy challenge in Germany, with 54.3% of the population reporting health literacy problems. This result pointed to challenges in navigating the health care system and understanding health-related information, with identified associations with factors such as age, migrant background, self-assessed social status, and functional literacy. The second Health Literacy Survey Germany study in 2020 [14] reinforced previous concerns with a further increased rate of individuals (58.8%) struggling considerably with health information. Even more so than in 2014, evaluating the trustworthiness of health-related information presented difficulties [15]. Up to 45% of the German population exhibit “problematic” or even “inadequate” internet skills [16]. Many individuals face so-called navigational needs. They depend on support from others to efficiently search and evaluate health information online [17]. As the amount of digital health information grows each year, the challenge of effectively navigating through this vast array of content becomes increasingly daunting, raising concerns about misinformation and health-related misconceptions.

During the work on the comprehensive GAP (Gut informierte Kommunikation zwischen Arzt und Patient, meaning “Well-informed communication between physician and patient” in English) project, which addressed evidence-based information [18-26], we studied the needs and requirements of internet users for health information online [18]. We developed criteria to assess web domains according to these needs. Assessing the quality of websites is nothing new. There are existing seals of approval, such as the afgis (Aktionsforum Gesundheitsinformationssystem eV, meaning “Action Forum Health Information System eV” in English) and Health On the Net Foundation Code of Conduct seals, which check whether the website operator meets certain transparency and quality standards. An afgis certificate is valid for 1 year. However, with Health On the Net Foundation Code of Conduct, there were several issues [27], and on December 15, 2022, the Health On the Net Foundation discontinued its services. Different seals potentially signify different standards of certification. Another issue with these seals is that the pages that have a seal cannot easily be found via a central search. To address these issues by centrally and consistently rating domains inside a search platform, we developed a search engine in 2020. This search engine was originally called “GAP search” and later renamed as “tala-med Suche” in German and “tala-med search” in English.

The topic of data retrieval in medicine is generally relevant and widely researched. Efforts ranging from decentralized search systems for patient data in registries [28] to tools such as Informatics for Integrating Biology and the Bedside (i2b2) [29] that allow the creation of cohorts, for which data security plays an important role [30], have been investigated. In addition, for public data, such as the Medical Subject Headings (MeSH), search capabilities play an important role [31], including the requirement for an intuitive user interface design [32]. In terms of scraping web content, which could be used to create a search platform, the Sampled German Health Web [33,34] created an index of German-language health-related web pages using a special focused crawler. The Sampled German Health Web index was restricted to pages from the top-level domains .de, .at, and .ch, and the domains were automatically filtered for health-related content while crawling using a support vector machine. By contrast, the new tala-med search engine, which was used in this study, relied on hand-picked, high-quality, German-language health websites. These domains—more than 50 of them—underwent a rigorous evaluation process. This evaluation was based on the categories *trustworthiness* (with the subcategories *authority*, *independence*, and *evidence based*), *recency*, *user-friendliness*, and *comprehensibility*. The ratings of these categories influence the order of the results on the SERP. The search engine also values user privacy by avoiding data collection and advertising. The detailed design of the search engine is described in the *Methods* section and in Multimedia Appendices 1-3.

By assessing the acceptance and usability of this new technology, we aimed to improve the implementation of the search tool. Numerous studies in the field emphasize the significance of outcome measures as crucial indicators of a technology’s effectiveness, user-friendliness, and potential for widespread adoption. User acceptance, often determined by perceived usefulness, ease of use, and individual attitudes, is critical for a technology’s long-term viability [35,36]. Moreover, the usability of a technology, including learnability, efficiency, and user satisfaction, significantly affects user experience and reduces possible adoption barriers [37]. Evaluating these dimensions can provide insight into how well technology meets users’ expectations, addresses their needs, and contributes to successful implementation. In the context of health care technology, this scrutiny is particularly relevant because effective tools for accessing medical information can significantly impact health care outcomes [17,38].

In this study, the acceptance and usability of our newly developed search engine was evaluated by means of a questionnaire study and actual use by tracking user behavior during the first months of operation.

### Related Works

The usability and accessibility of digital platforms are essential for ensuring that all users, including those with disabilities, can effectively engage with websites and mobile apps. Mateus et al [39] conducted a systematic mapping of accessibility issues, revealing that automated tests covered <40% of accessibility problems on websites and even fewer on mobile apps. The study stressed the importance of including users with disabilities in evaluations because user testing uncovered many issues missed by automated tools and expert inspections. This underscores the need for a comprehensive evaluation approach that combines expert and real-world user input to improve accessibility.

Petrie and Bevan [40] expanded on this by exploring the interplay between usability, accessibility, and user experience. The authors defined usability in terms of effectiveness, efficiency, and satisfaction, focusing on aspects such as learnability, flexibility, and safety. Importantly, they emphasized that accessibility is an integral component of usability and that digital systems should cater to the widest possible range of users, including those with disabilities. They also introduced the concept of user experience, which goes beyond usability to include users’ emotional responses and satisfaction with a system. This holistic approach is essential for creating digital platforms that are both functional and engaging for all users.

Belinda et al [41] provided a deeper look into the internal and external usability factors that affect website performance. The authors used automated tools such as GTmetrix and Website Grader to measure internal attributes such as performance, load time, and page size, while external attributes such as ease of navigation and user satisfaction were assessed through surveys. Their findings revealed that some websites performed well from a user perspective but were found lacking in technical performance, particularly in terms of load times and page requests. This highlights the need to address both internal and external usability factors to create well-rounded digital platforms.

Kritz et al [42] explored the online resources and tools used by European physicians to gather medical information. The authors found that physicians frequently relied on general search engines and faced significant barriers in accessing high-quality, trustworthy medical content. Medical specialists were more likely to use medical research databases, while general practitioners often faced barriers such as lack of time and language restrictions. The study highlighted the need for improved medical search tools tailored to the specific needs of different physician subgroups. Kritz et al [42] concluded that user-centered medical search tools could significantly improve accessibility and the quality of online medical information.

Strecker et al [32] also contributed to the usability of medical search tools, focusing on the MeSH Browser. The authors evaluated a newly developed multilingual MeSH Browser, which introduced improvements in user interface design to enhance the accessibility and usability of medical literature searches. The results showed that contemporary web design principles led to significant improvements in navigation and overall user satisfaction, further underscoring the importance of continual evaluation and enhancement of information systems in the medical realm.

Eysenbach and Köhler [43] studied how consumers search for and appraise health information on the internet. The qualitative study revealed that users, despite using suboptimal search techniques, were able to retrieve health information quickly. However, users rarely checked critical indicators of credibility, such as the “About us” sections or disclaimers, relying instead on superficial factors such as professional design and ease of use. This reliance on superficial credibility indicators poses risks, particularly in the health care field, where the accuracy of information is paramount. Eysenbach and Köhler [43] suggested that further research is needed to develop educational and technological tools that guide users toward high-quality health information.

Zhang [44] expanded the understanding of how consumers select sources for health information by identifying 5 categories of factors that influence source selection: source-related factors, user-related factors, user-source relationships, characteristics of the problematic situation, and social influences. The study also identified a range of criteria that mediate the influence of these factors on source-selection decisions, including accessibility, quality, usability, interactivity, relevance, usefulness, and familiarity. Zhang [44] concluded that a personalized approach to health information systems is necessary to provide effective access to health information because different consumers prioritize different factors when selecting sources. This insight strongly indicates the need for more personalized information services that cater to individual user preferences and needs.

In conclusion, the literature emphasizes the critical role of accessibility, usability, and user experience in the design of digital platforms, especially health care websites. While Saad et al [45] have highlighted general usability problems in health care websites, the studies by Kritz et al [42], Eysenbach and Köhler [43], and Zhang [44] focus on the challenges that users face in retrieving and assessing the credibility of health information online. A personalized and user-centered approach to the design of health care information systems would improve the accessibility and quality of health information, meeting the diverse needs of consumers and health care professionals alike [40,44].

## Methods

### Search Engine Development

#### Requirements

The functional requirements for the tala-med search engine included the ability to crawl and index health information websites, implement quality assessment filters, and provide search term suggestions and synonym handling. The nonfunctional requirements focused on maintaining user privacy through self-hosting, ensuring a user-friendly interface, and optimizing performance to handle large sets of synonym mappings efficiently.

#### Implementation

##### Design

To implement tala-med search, we used software with a self-hosting capability because of privacy considerations. Furthermore, modern technology with single-page application design using a web application programming interface was required to compete with modern search engines. Therefore, the selection available was limited. As no single product met all our requirements, we built a search engine stack ourselves, consisting of crawler, middleware, and front end, using existing components and established technologies.

##### Back End

We created the web index using the open-source crawler software Fess [46]. For boilerplate removal, we used Mozilla’s Readability tool to strip HTML tags and display core content, cleaning up crawled websites for our search index [47]. As middleware, we used Elastic App Search [48] (now part of Elastic Enterprise Search [49]), which relies on Elasticsearch. This software allows for setting up weighting mechanisms for the search equation (Multimedia Appendix 1) and configuring filters. In our case, a set of criteria for quality assessment were developed, which we integrated as filters. These quality assessment criteria were adapted from a systematic review by Eysenbach et al [6] that identified criteria used from 1969 to 2001 for evaluating the quality of health information online. We identified 74 criteria [18] to assess the content quality of different health-related websites and rigorously evaluated >50 German-language health information providers (Multimedia Appendix 2) using these criteria, assessing 1 main page and 5 randomly selected subpages per provider. To compile the list of relevant providers, we started with a list from a Bertelsmann study [3] and adapted it with the help of domain experts. The 74 quality criteria were later condensed into 4 categories: *trustworthiness*, *recency*, *user-friendliness*, and *comprehensibility*. The scores of these categories were added to the search index after boilerplate removal and divided into 4 value ranges reflecting the quality of each category, using 4 quantiles for differentiation. These scores served as quality indicators and filter categories, influencing the order of the results on the SERP. More details about the internal mechanisms of the search ranking can be found in Multimedia Appendix 1.

App Search supports the consideration of synonyms, which is crucial in medical language due to varied etymology and numerous abbreviations. Our goal was to enable the search engine to handle synonyms effectively. In App Search, sets of up to 32 synonyms can be created, allowing synonyms of a matched word to be considered in searches. We generated synonym sets by collecting word types and pairs from the document text, filtering against a German stop word list, resulting in approximately 6 million unique entries. These entries were matched against an experimental German interface terminology—SCT-GIT—linked to Systematized Nomenclature of Medicine–Clinical Terms (SNOMED-CT) [50], yielding approximately 40,000 mappings to SNOMED-CT codes. Synonym links in SCT-GIT were then used to add synonyms for each matching string, finding at least 1 synonym for approximately 30,000 SCT-GIT terms. We limited the number of synonym sets to 500 to maintain performance, using only sets with at least 4 synonyms, resulting in a median set size of 4.

##### Front End

**Figure 1:**
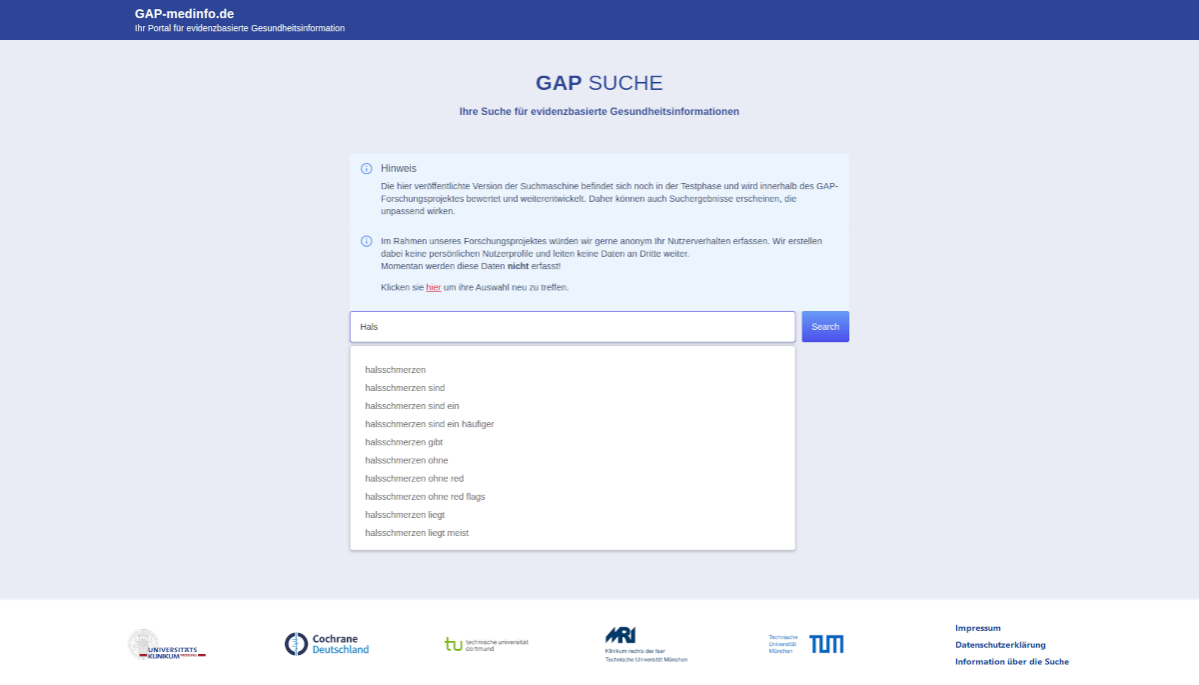
Landing page of the search displaying the initial search query field.

**Figure 2:**
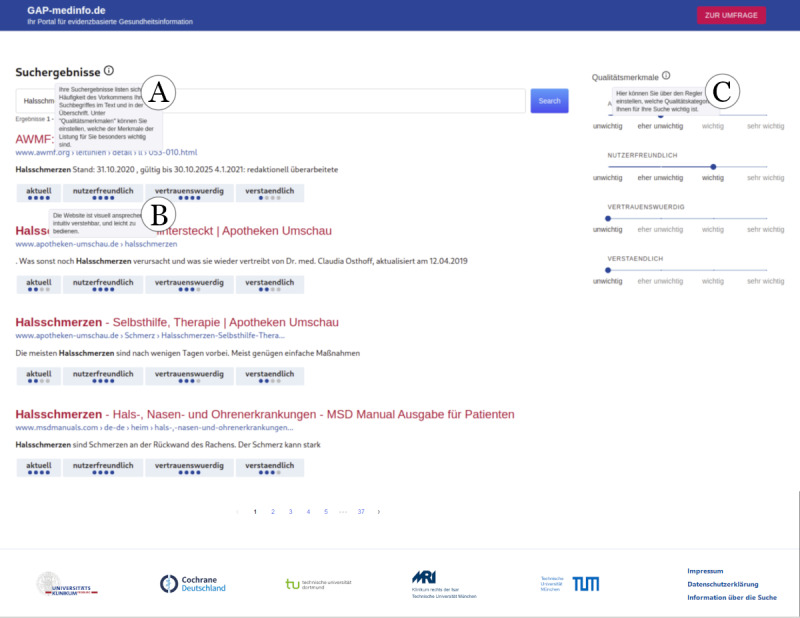
The search engine results page consists of 3 areas. (A) The search query. (B) List of search results. Each search result shows the quality in the 4 filter categories. (C) Slide controls allow users to set a threshold for each filter category (aktuell: recency, nutzerfreundlich: user-friendliness, vertrauenswürdig: trustworthiness, and verständlich: comprehensibility). In each of these areas, more information can be displayed by means of tooltips, as shown in the graphic (next to each label).

The front end was custom developed with a slim design inspired by Strecker et al [32], featuring search term suggestions and a user-friendly filter selection (Figure 1). After entering a search term, the interface displayed a SERP with evaluated German-language health information providers, showing their scores in the 4 filter categories through graphical indicators (Figure 2). Hovering over these graphics revealed explanations, and the bottom of the page included pagination and a footer with logos and links to pages displaying the imprint and data protection information.

##### Pilot Testing

The search engine was pilot-tested between October 2020 and the end of June 2021. Adapting to the challenges posed by the COVID-19 pandemic, the pilot testing format shifted from a face-to-face setting to a web-based setting with a small group of academic staff from the University of Freiburg. The evaluation, involving 12 participants, consisted of screen recordings of search tasks and a qualitative survey regarding user-friendliness. After the pilot tests, technical enhancements were implemented, guided by the qualitative insights. These findings formed the basis for the subsequent questionnaire study.

##### Availability

The current version of the search engine can be accessed on the web [51]. Please note that there have been some significant changes to the website structure since this study was conducted.

### Evaluation

#### Design

We used 2 data collection methods to evaluate user behavior. First, we asked users to fill out a questionnaire (self-perception). Second, we recorded user behavior via web tracking independently and unreferenced to the questionnaire (external perception). The study protocol was published in 2019 [22].

#### Questionnaire Study

The study used a cross-sectional design with data collected through a web-based questionnaire. The questionnaire was developed after pilot testing of the search engine and underwent an internal pretesting round before recruitment began.

#### Recruitment

The study used a recruitment strategy aimed at enrolling 200 participants. The first rounds of recruitment, starting in October 2020, which involved sending email invitations to local health initiatives, professional networks for physicians, and personal contacts, were unsuccessful in obtaining the desired number of participants. Participants were then recruited from the WisoPanel, an established online panel of German-speaking individuals [52]. The panel provided a pool of 14,900 potential respondents who had previously expressed an interest in participating in research studies. The survey was open for anyone accessing the link, but only participants from the panel (>70% of the data) were included in the analysis of this study to maintain a credible sample. Recruitment via the panel took place in May 2021. The survey ended at the end of May 2021. Panel members were invited to participate in the study via email. The email informed participants about the project and the aspects distinguishing the new search engine from other platforms.

To ensure that participants had firsthand experience with the platform before proceeding to the survey, they were asked to test the search engine first. We recommended that participants carry out 1 or 2 searches and familiarize themselves with the SERP and the features and functions of the website.

#### Survey Administration

The hyperlink that led participants to the search engine was sent to the panel members by email. Subsequently, the link to the survey was displayed as a pop-up element after 10 to 20 seconds of interaction with the search engine. Throughout the assessment phase, a button to access the survey was strategically positioned in the top right corner of the website to allow a direct link to the survey interface. No registration was required to complete the survey, ensuring seamless and voluntary participation. Participants had the option to cancel their responses at any time, and there were no time restrictions regarding the completion of the questionnaire. Back buttons allowed participants to review their answers before submitting the survey. Conventional technical methods, such as cookies, were used to prevent visitors from submitting their survey more than once.

The web-based survey was conducted using the Unipark tool [53], with the survey page seamlessly embedded into the search page via a modal window. After inviting panel participants, the survey remained accessible for 11 days and closed at the end of May 2021.

Before filling out the survey, participants were provided with an introductory text outlining the anticipated time commitment, project objectives, details about anonymity, the option to terminate the survey at any point, data protection measures, and contact information for queries. To proceed, participants were required to confirm their understanding by checking a box and consenting to the specified use of their data (informed consent). No personal data were collected in the survey, and participants were instructed not to provide any identifying information in their open responses. Access to the data was restricted to members of the institutions participating in the study.

#### Survey Characteristics

On the basis of established scales (the German version of the self-assessment eHealth Literacy Scale [G-eHEALS] [54] and the System Usability Scale [55] adapted by Quirmbach [56] and Magin et al [57]), we developed a 25-item questionnaire across five dimensions: (1) sociodemographic data; (2) internet use; (3) digital health literacy; (4) usability, acceptance, and innovative aspects of the search engine; and (5) search filters. The final questionnaire contained 24 single-choice questions and 1 open-ended question (Tables S1 and S2 in Multimedia Appendix 4). The items in the questionnaire were not randomized. No form of adaptive questioning was used. All questions, except for the open-ended question, were mandatory; nonresponse options were not provided. Thus, there was no additional consistency or completeness check before submission. The survey took approximately 5 minutes to complete.

#### Statistical Analysis

Descriptive statistics were used to summarize the characteristics of the study participants, including age, gender, occupation, educational level, and internet use for health information, as well as self-perceived digital health literacy. Only completed questionnaires were analyzed. No statistical correction was applied to adjust the sample. To assess the acceptance and usability of the search engine, Likert-scale items related to these dimensions were combined to create scale values. The acceptance scale measured participants’ willingness to use the search engine again and to recommend it to others. The usability scale evaluated participants’ agreement with statements related to comprehensibility, clarity, the effectiveness of the search, confidentiality, advertising, the ease of learning, and using the search engine.

One reviewer analyzed the content of the open-ended question and assigned the comments to different categories inductively.

Multiple regression analyses were conducted to explore predictive factors influencing the acceptance of the search engine. The factors tested in the regression analyses included age, gender, occupation, educational level, the frequency of internet use for health information, and self-perceived digital health literacy.

#### Reporting

This study is reported based on the guidelines of the Checklist for Reporting Results of Internet E-Surveys (CHERRIES) [58].

#### User Behavior Tracking

Behavior tracking data were collected from all consenting users accessing the search engine between October 2020 and June 2021 using the web analytics software Matomo (Matomo.org) [59]. Matomo is a powerful open-source web analytics platform designed to help website owners and organizations gain insights into their online presence while respecting user privacy. Its primary purpose is to track website traffic by tracking website visitor behavior. What sets Matomo apart from services such as Google Analytics is its commitment to data privacy and General Data Protection Regulation (GDPR) compliance. Matomo allows users to maintain control over their data by hosting the data on their own servers, ensuring that sensitive information is not shared with third parties. Matomo is easy to integrate into web pages and can also be customized to track specific custom data. Ripp and Falke [60] successfully used Matomo to track user behavior in their research project, where they analyzed search behavior for certain keywords on the online information system Grammis.

We installed Matomo on our own server and integrated the tracking into our search engine’s front end. We enabled the tracking to record the filter settings and capture the search term beyond the standard visit data, such as how long users spent on the website, and which of the results they clicked on. Structurally, Matomo records several actions for each visit, containing the duration among other data (Figure S1 in Multimedia Appendix 5). However, for the last action of a visit, the duration is not recorded. We have filled the empty value with the median of all actions from the particular visit to obtain a more realistic value for the entire visit duration.

Only data from consenting users was recorded and subsequently descriptively analyzed for the 9-month time period. Therefore, this sample differs from the sample of the questionnaire study but may also include users who completed the questionnaire. The data processing for the data analysis was realized with a Python script directly accessing Matomo’s database using specific SQL queries.

### Ethical Considerations

The study was approved by the institutional review board at the University of Freiburg (Ethikkomission-Freiburg 559/17) as an extension to the GAP study [22], and a document on data protection was agreed upon. As an incentive, the questionnaire study participants could take part in a prize draw for 1 of 25 vouchers to a bookstore worth €20 (US $24.33) after completing the survey.

## Results

### Questionnaire Study

#### Participants

During the time the survey was open (from October 2020 to the end of May 2021), there were 1577 unique site visitors to the search engine website, with 1426 (90.4%) visitors to the first survey page. Of these 1426 visitors, 1250 (87.7%) agreed to participate in the survey. Of these 1250 participants, 1123 (89.8%) completed the survey.

Of the 1123 survey respondents, 802 (71.4%) were from the panel described in the Recruitment subsection. The age distribution of these participants was approximately normal, with a little more than a quarter of the participants (215/802, 26.8%) falling into the age category of 50-59 years. The gender distribution was also fairly balanced between male and female, with a little more than half of the respondents (432/802, 53.9%) self-identifying as female. Regarding occupational background, the majority of the participants (726/802, 90.5%) did not work in a medical profession.

Educational levels were notably higher than in the general population due to recruitment through the scientific panel, with 69.3% (556/802) of the participants having completed upper secondary education (at least level 3 in the International Standard Classification of Education [ISCED]-2011) and 46.3% (372/802) holding a bachelor’s or master’s degree or higher (ISCED-2011 levels 5-8).

The study participants recruited from the web-based panel reported searching for health information online more frequently than the general population, with 65.4% (524/802) searching several times a month at the minimum and 27.7% (222/802) searching at least several times a week. Only 1.5% (12/802) of the participants indicated that they “never” searched the internet for health information.

Participants’ self-perceived digital health literacy was measured by the G-eHEALS items. Of the 802 participants, 291 (36.2%) felt confident in making health-related decisions based on internet information, while 461 (57.5%) felt capable of distinguishing reliable from questionable information online.

More information about the participants is available in Table S3 in Multimedia Appendix 4.

#### Acceptance and Usability

**Figure 3:**
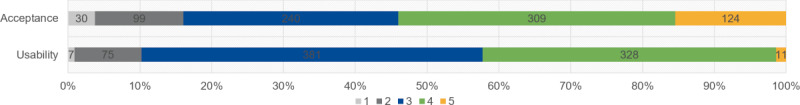
Scale values for acceptance and usability (n=802). The Likert-scale response options were as follows: 1=strongly disagree, 2=disagree, 3=partially agree, 4=agree, and 5=strongly agree. There was one negative question, which was adjusted for calculating the scale values.

Scale values were created by combining items relating to the acceptability and usability of the search engine (Figure 3).

The results show agreement regarding the acceptance and usability measures of the new search engine (Figure 3 and Table 1). In particular, more than half of the participants expressed their willingness to use the search engine again (465/802, 58%) and to recommend it to others (507/802, 63.2%). In addition, 79.3% (636/802) of the respondents agreed or strongly agreed that using the search engine was quick to learn, and 77.3% (620/802) to 98.6% (791/802) agreed at least partially with other statements related to usability aspects.

**Table 1:** Detailed items for acceptance, usability, and innovative aspects (n=802). The Likert-scale response options for acceptance and usability were as follows: 1=strongly disagree, 2=disagree, 3=partially agree, 4=agree, and 5=strongly agree. The Likert-scale response options for the innovative aspects item were as follows: 1=very unimportant, 2=unimportant, 3=neither unimportant nor important, 4=important, and 5=very important.

Content		Scores, mean (SD)
**Acceptance**		
	Item 1: potential reuse	3.58 (1.02)
	Item 2: recommendation to others	3.68 (1.03)
**Usability**		
	Item 1: quick to learn	4.03 (0.86)
	Item 2: efficient scannability of the SERP^a^	3.63 (1.00)
	Item 3: clarity of the SERP	3.75 (1.00)
	Item 4: functions *not* comprehensible^b^	2.37 (1.29)
	Item 5: appreciation for the absence of advertising	4.66 (0.66)
	Item 6: functionality meets expectations	3.45 (1.03)
	Item 7: careful handling of personal data	3.59 (0.88)
	Item 8: no commercial bias	3.55 (0.95)
	Item 9: fast access to relevant information	3.77 (0.99)
	Item 10: innovative approach	3.58 (1.03)
Innovative aspects of the search engine (anonymous searches)		4.50 (0.76)

^a^SERP: search engine results page.

^b^Item 4 (comprehensibility of functions) was a negated question.

Users particularly appreciated the absence of advertising on the platform, with 93.1% (747/802) agreeing or strongly agreeing that the absence of advertising was a significant benefit. The search engine’s user-friendly interface and the ability to adjust search results through the use of filters were also well received by participants. Furthermore, the unique features of the search engine, notably the anonymous searches without the creation of user profiles and independence from sponsors, received high importance ratings from 91.3% (733/802) of those surveyed.

More detailed results are available in Tables S4-S6 in Multimedia Appendix 4.

Of the 802 participants, 561 (70%) to 622 (77.6%) indicated that they used the filter functions that could be enabled to change what was displayed on the SERP (Table 2; Figure 2). Furthermore, 92.3% (518/561), 93.9% (553/589), 94.7% (567/599), and 96.5% (600/622) of those users who used the filters agreed at least partially that the filter functions were helpful in finding trustworthy, recent, user-friendly, and comprehensible results, respectively. The *recency* filter was particularly well received, with 46% (286/622) of the users strongly agreeing that it was helpful. More detailed results are available in Table S7 in Multimedia Appendix 4.

**Table 2:** Helpfulness of filters (n=802). The Likert-scale response options were as follows: 1=strongly disagree, 2=disagree, 3=partially agree, 4=agree, and 5=strongly agree.

Content			Participants, n (%)^a^		Values, mean (SD)
**Helpfulness of filters for each filter**					
	Item 1: trustworthiness	599 (74.7)		4.11 (0.92)	
	Item 2: recency	622 (77.6)		4.25 (0.84)	
	Item 3: comprehensibility	589 (73.4)		4.05 (0.93)	
	Item 4: user-friendliness	561 (70)		3.91 (0.93)	

^a^All other participants answered, “I did not use this function, I cannot evaluate this question.”

**Figure 4:**
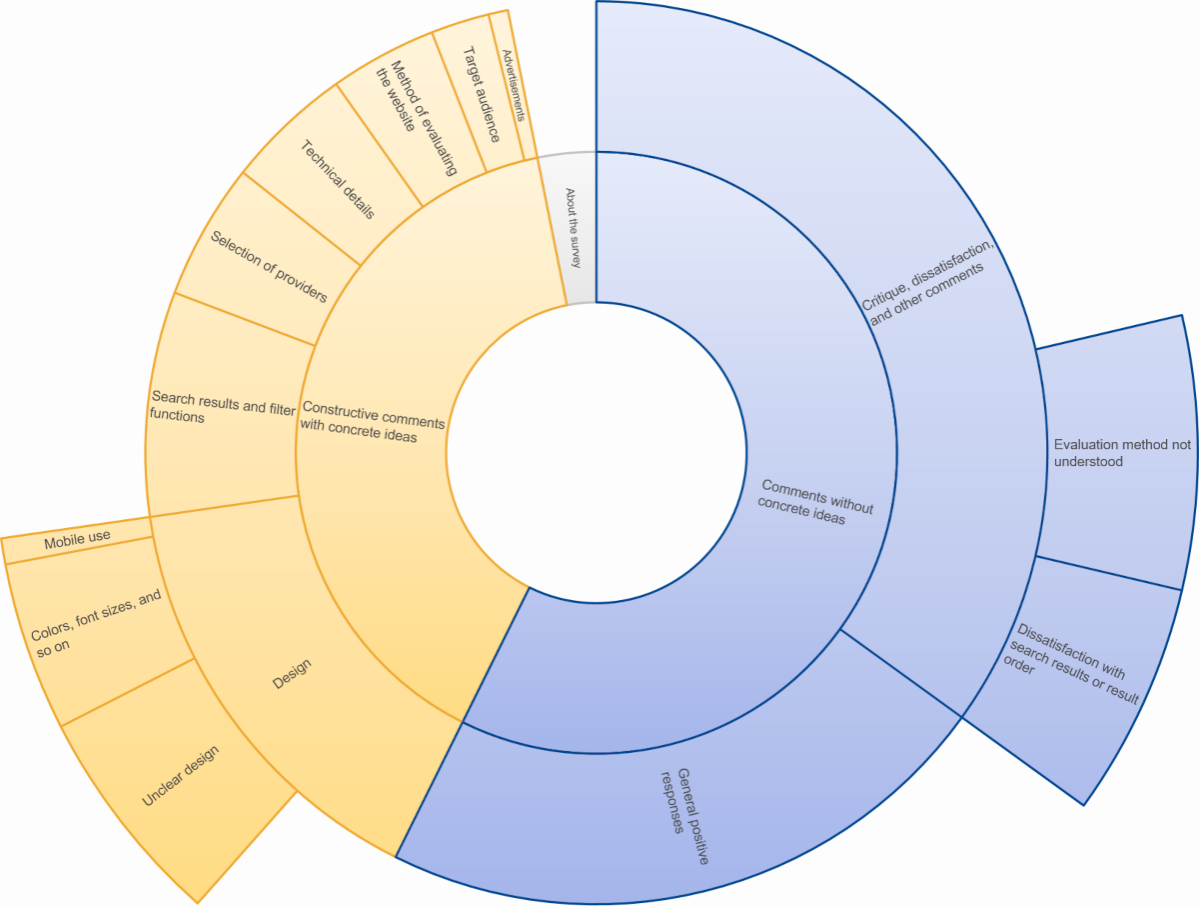
In the analysis of 263 comments in response to the open-ended question, 10 main themes were identified inductively: design; search results and filter functions; selection of providers; technical details; method of evaluating the website; target audience; text on the website; comments about the survey; general positive responses; and critique, dissatisfaction, and other comments. The illustration shows a hierarchical visualization, with the size of the elements indicating the quantity of comments.

In the open-ended question, which aimed to supplement the data with qualitative results, participants were asked to share their comments and suggestions for improvements. Of the 802 participants, 263 (32.8%) replied to the question. Multiple aspects were mentioned. In an inductive approach, 10 main themes were identified (Figure 4). Many of the participants commented on the usability aspects of the website. Of the 236 comments, 76 (32.2%) included constructive criticism and specific suggestions for improving the website in the future. Many commenters made suggestions for improving the design because they found some aspects unclear or overwhelming. Others did not fully understand the methodology behind the evaluation of the selected providers or the reason why only specific providers, including commercial web pages, were shown on the results page and asked for more clarification. Some commenters were happy with the site as it stood and did not ask for any changes, while some conveyed disappointment with the results they discovered on the SERP.

#### Predictors of Acceptance

We conducted multiple regression analyses to examine the predictive factors influencing responses to the acceptance scale. Age, gender, occupation, educational level, frequency of internet use for health information, and self-perceived digital health literacy were tested as influencing factors. All factors were first tested individually and subsequently tested simultaneously due to the potential error introduced by multiple testing. Overall, only minor discrepancies were identified. Significance levels were set at *P*<.05. Table 3 presents more details.

**Table 3:** Predictive factors for acceptance (n=802). Categorical variables were dummy coded.

Predictive factors for acceptance		Individual testing of each predictor									Simultaneous testing of all predictors^a^						
		B (SE)	*F* test (*df*)		*P* value		*R*^2^ (%)		B (SE)			*F* test (*df*)		*P* value		η_p_^2^ (%)	
Age		*0.07*^b^ (*0.02*)	*7.97 (1)*		*.005*		*0.99*		*0.07 (0.03)*			*7.27 (1)*		*.007*		*0.91*	
**Gender**				0.04 (2)		.96		0.01					0.20 (2)		.82		0
	Diverse	0.01 (0.49)	0.00 (1)		.99				−0.07 (0.49)			0.02 (1)		.89			
	Female	0.02 (0.07)	0.09 (1)		.77				0.04 (0.07)			0.35 (1)		.55			
	Male	Reference	Reference		Reference		Reference		Reference			Reference		Reference		Reference	
**Occupation**				1.82 (2)		.16		0.45					1.14 (2)		.32		0.29
	Medical doctor	0.12 (0.12)	0.92 (1)		.34				0.07 (0.12)			0.28 (1)		.59			
	Other medical profession	−0.56 (0.35)	2.62 (1)		.11				−0.49 (0.35)			1.93 (1)		.17			
	Not working in a medical profession	Reference	Reference		Reference		Reference		Reference			Reference		Reference		Reference	
**Highest level of educational attainment**				*2.57 (4)*		*.04*		*1.27*					1.55 (4)		.19		0.78
	No formal secondary education (ISCED^c^ levels 0-1) or lower secondary education (*Hauptschulabschluss*, ISCED-2011 level 2)	*0.51 (0.19)*	*6.81 (1)*		.009				*0.41 (0.20)*			*4.22 (1)*		.04			
	Lower secondary education (*Realschulabschluss*, ISCED-2011 level 2)	0.33 (0.17)	3.85 (1)		.05				0.26 (0.17)			2.33 (1)		.13			
	Upper secondary education (*Fachabitur oder Abitur*, ISCED-2011 levels 3-4)	0.16 (0.17)	0.94 (1)		.33				0.13 (0.17)			0.58 (1)		.45			
	Tertiary education: bachelor’s or master’s degree or equivalent (ISCED-2011 levels 6-7)	0.21 (0.16)	1.67 (1)		.20				0.17 (0.16)			1.14 (1)		.29			
	Doctorate degree (ISCED-2011 level 8)	Reference	Reference		Reference		Reference		Reference			Reference				Reference	
Frequency of internet use for health information		*0.15 (0.04)*	*17.16 (1)*		*< .001*		*2.1*		*0.13 (0.04)*			*11.98 (1)*		*<.001*		*1.5*	
Self-perceived digital health literacy: information search		*0.12 (0.04)*	*9.69 (1)*		*.002*		*1.2*		0.08 (0.05)			3.03 (1)		.08		0.38	
Self-perceived digital health literacy: information assessment		0.05 (0.04)	1.79 (1)		.18		0.22		−0.01 (0.05)			0.04 (1)		.85		0	

^a^Variance resolution of simultaneous testing: *R*^2^=5.02%.

^b^Factors with significant predictions (*P*<.05) are shown in italics.

^c^ISCED: International Standard Classification of Education.

There were 2 significant results in predicting the acceptance of the search engine. First, the acceptance rating increased with age, rising by 0.07 scale points (B=0.07) for each additional 10-year age group. Second, the ratings for acceptance were higher among participants with more frequent internet use for health information.

The factors “highest level of educational attainment” and “self-perceived digital health literacy” only showed significant correlations with the acceptance ratings in the individual tests and slightly lower correlations in the simultaneous tests. Participants with lower levels of education showed stronger acceptance ratings than those with higher levels of education. For this predictor, the reference category was set as “doctorate degree” (ISCED-2011 level 8). Acceptance ratings among persons without any formal secondary education (ISCED-2011 levels 0-1) or with a lower secondary education (*Hauptschulabschluss*, ISCED-2011 level 2) were 0.41 scale points higher than among persons with a doctorate degree (B=0.41). However, if we examine the effect strength using partial eta–squared (η_p_^2^), only small effects were shown overall for all factors (1%=small effect, 6%=medium effect). No significant results were found for the factors *gender* or *occupation*.

### User Behavior Tracking

**Figure 5:**
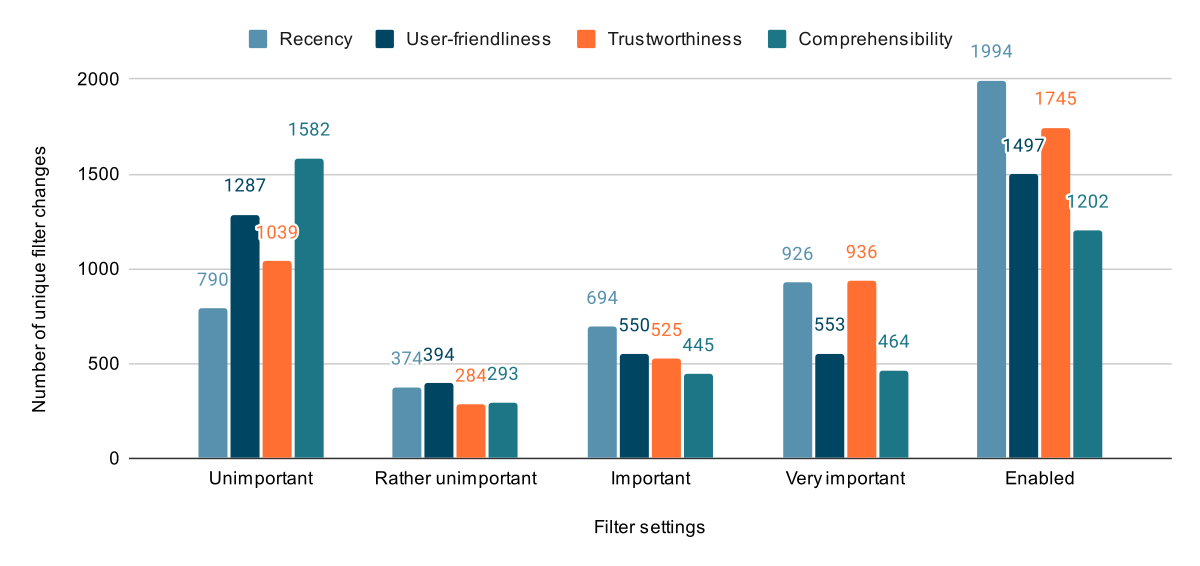
Distribution of the filter settings of the 2784 nondefault unique filter changes across filter categories. We consider a filter category to be “enabled” if a filter category is not set to “unimportant.”.

For a period of 9 months (from October 2020 to the end of June 2021), user behavior of consenting users was tracked using Matomo. The main aspects recorded were the search queries and the use of the filter functions. The data indicated that 1631 visitor sessions had taken place, with 3090 searches using 1984 different search terms across 1924 visits. The search terms had an average lexical token count of 1.74 (SD 1.14). Of the 1984 search terms, 1096 (55.24%) had a lexical token count of 1, while 888 (44.76%) had a lexical token count of >1. The maximum lexical token count was 13. We observed that 28.9% (893/3090) of the search queries were conducted through mobile devices such as smartphones and tablets. On average, visitors conducted 1.64 (SD 1.31) searches per visit, with an average duration of 137.45 (SD 278) seconds and a median duration of 49 (IQR 24-119) seconds. The top 3 search terms were “corona,” “rückenschmerzen” (back pain), and “husten” (cough; Table S1 in Multimedia Appendix 5). Each search term was queried 1.57 (SD 3.46) times on average; of the 1984 search terms, 1621 (81.7%) were entered just once. Users could personalize the order of the displayed search results, depending on the prioritization of the different filter categories (*trustworthiness*, *recency*, *user-friendliness*, and *comprehensibility*), all of which were initially set to “unimportant” by default. Within the search interactions, overall, 47,532 actions were performed, among which were 1358 (2.86%) outlink actions (ie, external domains clicked by website visitors) and 34,490 (72.56%) filter changes in total. The high number of filter changes was due to the fact that the filters were often switched back and forth in a search, which led to multiple occurrences of the same filter settings. This behavior was probably observable due to the fact that, given the limited corpus, the result set could be empty for certain search terms in combination with a narrow filter setup; therefore, it was necessary to change back the filter to obtain results. Excluding these repeated changes, of the 34,490 filter changes, 5534 (16.05%) were unique. In addition, after removing the initial filter setup where all categories were set to the default value, we ended up with 2784 nondefault unique filter changes used in 15.37% (475/3090) of the searches. *Recency* was used the most, followed by *trustworthiness*, *user-friendliness*, and *comprehensibility* (Figure 5). Consistent with the results on the usefulness of the filter function from the questionnaire study, the user behavior data showed that the filters for *recency* and *trustworthiness* were adjusted most frequently.

## Discussion

### Principal Findings

In this study, we evaluated the independent, noncommercial tala-med search for German-language medical information, placing a primary focus on transparency, independence, and evidence-based information. Our questionnaire study and user behavior tracking provide insights into the acceptance and usability of this innovative platform.

The survey resulted in a mean acceptance score of 3.63 (SD 0.98) and a mean usability score of 3.76 (SD 0.61) on a scale of 1 to 5 (1 signifying strong disagreement and 5 signifying strong agreement with acceptance and usability statements). Given that our user interface is similar to that of popular search engines such as Google and Bing and is advertisement-free, it is unsurprising that it was well received. However, there is still a need for optimization to achieve a higher reuse potential and a greater willingness to recommend the platform. Within the usability measures, item 1, which reflected the learnability of the search, achieved a high mean value of 4.03 (SD 0.86; Table 1). In terms of the effectiveness and clarity of our design (refer to items 2, 3, and 9 in Table 1), we still see room for improvement but are satisfied with the results for the initial release. Our findings align with those of Petrie and Bevan [40], who emphasized that usability, particularly learnability, is crucial for ensuring the platform’s effectiveness and user satisfaction. Similar to the study by Petrie and Bevan [40], our study highlights the need to continually evaluate and refine digital systems to cater to user needs and improve overall usability. The survey also revealed that the filters were used by >70% of the 802 users. Among these users, the filters were highly accepted on average, which matches with the tracking statistics revealing the high use of the filter functionality. The most used filter was *recency*, followed by *trustworthiness*, matching the survey outcome, which asked about the helpfulness of the filters. This was not the case for the 2 remaining filters. Although the filter for *user-friendliness* was used more often in our tracking data than the one for *comprehensibility*, it was perceived as less helpful than comprehensibility. In the future, we might inspect these filter categories in more detail and potentially improve their estimation. The insights support our decisions, including basing the design of our search engine on the insights of Strecker et al [32], which demonstrated how improvements in user interface design could lead to higher user satisfaction. The high acceptance of the filters for *recency* and *trustworthiness* in our study also echoes the findings of Strecker et al [32], who highlighted that content navigation and relevance contribute to overall user satisfaction. Nevertheless, the insights encourage us to improve and possibly expand the filters and the design. The fact that our search engine does not share any data with partners or advertisers and does not record the visitor’s behavior to create profiles was rated as very relevant, with a mean score of 4.5 (SD 0.76). This result confirms the importance of our general endeavor driven by data privacy and noncommerciality as our fundamental motivations for creating the search engine. This finding is consistent with the work of Eysenbach and Köhler [43], who found that while internet users claimed to prioritize credibility when assessing health information online, they often relied on superficial factors such as website design and ease of use, rather than more reliable indicators such as the “About us” sections or disclaimers. Our platform addresses these shortcomings by offering more explicit filters, such as *trustworthiness*, which help users find credible information without having to rely solely on superficial design elements. In our calculations on the predictors of acceptance, it can be concluded that while the effects may be small, the search engine was particularly well accepted among older users, users with a high frequency of internet use for health information, and those with lower levels of education. This finding suggests that the new search engine may effectively cater to segments of the population with lower health literacy and digital health literacy and greater requirements for navigational assistance, bridging the gap in accessing reliable medical information online. As discussed by Kritz et al [42], general practitioners and medical specialists often face barriers in accessing high-quality medical content due to limited time and the overwhelming amount of information online. Our search engine’s focus on evidence-based information and user-friendly design helps mitigate these issues, making it a suitable tool for both health care professionals and the general public.

This reinforces the necessity to consider navigational needs in health information platforms. Digital health literacy has improved in recent years, particularly among persons with low levels of education [14]. The ability to assess health information has also improved over time and over the course of the COVID-19 pandemic. Digital health literacy improved for younger but not for older people over the course of the pandemic [15]. Even with some of these improvements, health literacy is still low within the German population and particularly among people with low levels of education. This observation aligns with the findings by Zhang [44], who identified that source selection for health information is influenced by a wide range of factors, including accessibility, quality, usability, and personal relevance. Our platform’s appeal to older users and those with lower health literacy supports the conclusion drawn by Zhang [44] that a personalized approach, considering different user-related factors, is crucial for effectively meeting the diverse needs of consumers when they access health information. The correlation between low educational levels, low social status, higher age, and lower health literacy rates has become even stronger [14]. This emphasizes the need to address low health literacy to improve social inequities. The new search engine attempts to address this need by assessing health information online and by making high-quality information easily searchable.

Many of the comments highlight the fact that at the time of the survey, participants used the first version of the search engine, which still had some technical and appearance-related weaknesses. Since the survey was conducted, a number of revisions have been made, some of which correspond to suggestions made in the comments, such as adding more information about the principles of the quality assessment and the functions of the websites and removing certain commercially funded information sources.

The platform’s emphasis on anonymous searches and independence from advertising and sponsors resonated especially well with the participants, underscoring the importance of developing alternatives to commercial search platforms. The usability of our platform, free from external advertising influence, aligns with the findings by Belinda et al [41], who evaluated both internal factors (such as page load time and performance) and external factors (such as ease of navigation and organization of information) as critical to the overall user satisfaction with websites. Our platform similarly emphasizes ease of use and efficient performance, which were key contributors to its positive reception. The general results align with previous findings on the importance of user-friendly interfaces [61-63] and the impact of advertising on users’ perceptions [64].

### Limitations

#### Limitations of the Technology

Some participants expressed dissatisfaction with the displayed results. Several respondents found the design of the SERP and individual results confusing due to an overload of information in one place. Potential design changes have been suggested to improve clarity. Currently, some search results are duplicated on the results page. To enhance the appearance of the SERP, it is desirable to deduplicate the search index. This would mean displaying more relevant information and fewer distracting results. To address the fact that sometimes no results were displayed due to strict filter settings, we could display results with nonexact matches for the filter category score below the exact matches and visually indicate them.

Participants encountered difficulties in understanding how the evaluation process worked. Efforts should be made to make the evaluation process easier to understand when using the website. Some improvements have been made over time, such as providing brief explanations of the filter categories when hovering over the graphic showing the score for each domain.

In addition, further clarification is necessary regarding the domains chosen, which comprise both independent and commercial providers. Over time, certain websites were excluded from being displayed on the site because of the conflicts of interest of their publishers (such as the site “Zentrum der Gesundheit” and a site funded by a pharmaceutical company). Currently, the website no longer displays commercial providers.

#### Limitations of the Questionnaire Study

While the questionnaire study provides valuable insights into the acceptance and usability of the new search engine, several limitations should be acknowledged. First, the recruitment methodology, relying on web-based panel recruitment, unintentionally led to an overrepresentation of individuals with higher educational levels than the general population and potentially more frequent internet use due to the online recruitment process. Among our participants, 46.3% (371/802) had completed tertiary education compared to only 18.5% of the general German population with a university degree or equivalent [65]. Similarly, 65.4% (524/802) of our participants reported searching for health information only at least once a month, while a Bertelsmann Foundation 2018 study indicated that only approximately 50% of the general German population seek such information at least once a month [2]. This potentially influences acceptance and usability ratings. Participants’ self-perceived digital health literacy, as measured by the G-eHEALS items, approximately aligned with that of the sample of the validation study of the German version of the questionnaire by Söllner et al [54]. However, this information should be viewed with caution because Kim et al [66] discovered a discrepancy between self-assessments and actual ability. Actual ability was not assessed in our study.

Second, as some of the participants only interacted with the search engine and its features for a brief duration, this short testing time proved to be a limiting factor, hindering the formation of comprehensive first impressions. The responses to the open-ended question suggest that some participants tested the features of the search engine for only a few seconds. This was caused by the pop-up window linking to the questionnaire opening after interacting with the website for 10 to 20 seconds. Such a brief interaction period was likely insufficient for users to thoroughly explore and understand the functionalities and benefits of the search engine. Consequently, the feedback provided by users on the acceptance and usability of the platform may not accurately reflect its true potential. The limited duration of testing may lead to superficial evaluations, whereby participants may base their judgments on initial impressions rather than informed use. This can result in an inaccurate assessment and therefore impact the results presented for the acceptance and usability measures. More extended testing periods could offer a more accurate assessment of the platform’s acceptance and usability over time. Furthermore, the implementation of controls for the length of the testing period could provide more reliable data, thereby ensuring that all participants have sufficient time to engage with the platform before completing the questionnaire.

#### Limitations of the User Behavior Tracking

As Matomo does not record the last action, we decided to substitute this missing value with the median of all actions from the particular visit. However, a more optimal solution would be to record of the last action, which could be triggered when closing the browser, but Matomo does not support this feature.

Unfortunately, due to data protection regulations, we were unable to link the search behavior with the survey data. It would have been interesting to see to what extent the search behavior and the questionnaire would have provided more information.

### Future Work

#### Further Evaluation of the New Search Engine

As these results are based on the first impressions of a group of users, data on long-term use and satisfaction have yet to be compiled. Future work should therefore address the need to evaluate long-term acceptance and usability measures of the site using a longitudinal study design. This could provide valuable insight into whether users actually use the search engine when they have an acute need to seek out medical information and whether they return to the site over time.

Future research should also consider comparing the search engine with other existing and established platforms to gain a deeper understanding of its unique advantages. Such investigations might involve participants executing identical search queries on both the novel search engine and other platforms and sharing their impressions of each experience, similar to the study by Strecker et al [32]. Particular attention should be given to participants’ abilities to assess the quality and reliability of the information found. Furthermore, a comparison of the specific results retrieved from both search engines could be undertaken, examining both the congruence and discrepancies in the results.

#### Further Improvements to the Search Engine

Ongoing revisions and updates to the search engine, including integration on partner websites, hold promise in expanding its reach and use, ensuring that it remains relevant and effective in addressing users’ health information needs. Since the data collection for this study was completed, there have been several revisions to the website.

As the qualitative survey showed, some users found the SERP complicated. To address this issue, a potential solution could be a clearly linked help page with a video explanation. Another solution could be to provide an animated assistant that guides users through the page.

One major disadvantage of the search engine in its current state stems from the fact that the evaluation of the domains—more than 50 of them—according to the quality criteria was performed in 2020, and there has not been any update to the evaluations thus far. The domains’ quality may have improved or declined in the time since the evaluation. A continual process with constant reevaluations of the domains is necessary to ensure an up-to-date rating of the domains accessible through the search engine. As we currently do not have the capacity to perform another evaluation or to establish a continual evaluation process for the domains, we have temporarily disabled the display of the filter category scores and the option to adjust the results based on these scores because the scores do not reflect the actual quality of the domains in their current state.

Thus, in future, inspired by Zowalla et al [34], we intend to evaluate various criteria automatically. In the study by Zowalla et al [34], the readability of the language was analyzed supported by a support vector machine. We plan to use more sophisticated approaches that also model contextual context. Therefore, we plan to create classifiers based on Bidirectional Encoder Representations from Transformers (BERT) [67]. We have in mind the domain-specific models GerMedBert [68] and BioGottBERT [69], the latter being a model of the German monolingual Robustly Optimized BERT Pretraining Approach (RoBERTa) [70] model GottBERT [71] specialized on medical language. A further improvement might be the enrichment of the texts with MeSH terms, similar to PubMed. Such approaches would enable us to process and rate documents individually, rather than evaluating domains using random samples.

From a technological standpoint, there are still notable areas for enhancement of the middleware, which could be implemented in the future. As App Search is proprietary and monolithic, to make it customizable and more extensible, we are currently exploring potential improvements. In particular, we are considering alternative technologies, as used by Scheible et al [31]. This would enable us to add features based on modern technologies. Specifically, we are contemplating a different approach to computing synonyms. Instead of using discrete structures, we plan to train and use a FastText model [72].

### Conclusions

The development of the independent, noncommercial tala-med search marks a step toward improving access to reliable and evidence-based health information online. By prioritizing transparency, independence, and high-quality content, the platform has the potential to bridge the gap in health information accessibility, especially for older and less-educated individuals. This study achieved its objective of evaluating the acceptance and usability of the platform, using user questionnaires and behavior tracking, and the results have been encouraging. The search engine was well received, with users appreciating its advertising-free design and filtering system, which addresses the navigational needs of individuals with lower health and digital health literacy.

User feedback also highlighted areas for improvement, particularly in the clarity of the design and filter functionality. Nevertheless, the platform’s emphasis on anonymous searches and independence from advertising resonated strongly with participants, reinforcing the importance of alternatives to commercial search engines. These findings are based on initial impressions, with long-term use and satisfaction data still to be collected. Future research should compare the search engine with existing platforms to further explore its unique advantages, while ongoing updates and potential integration on partner websites will enhance its reach and relevance.

In conclusion, the tala-med search demonstrates a promising step toward enhancing health literacy and empowering individuals to make well-informed health decisions. By addressing users’ navigational needs and promoting equitable access to high-quality, evidence-based medical information, the search engine has the potential to positively impact health literacy, reduce health disparities, and promote patient empowerment in the digital age.

## Appendix

Multimedia Appendix 1 Search engine development: search equation and filter estimation.

Multimedia Appendix 2 Search engine development: list of domains.

Multimedia Appendix 3 Search engine development: criteria evaluated.

Multimedia Appendix 4 Questionnaire study: questionnaire and detailed results.

Multimedia Appendix 5 User behavior tracking details.

## Abbreviations

afgis: Aktionsforum Gesundheitsinformationssystem eV (Action Forum Health Information System eV)
BERT: Bidirectional Encoder Representations from Transformers
CHERRIES: Checklist for Reporting Results of Internet E-Surveys
GAP: Gut informierte Kommunikation zwischen Arzt und Patient (Well-informed communication between physician and patient)
GDPR: General Data Protection Regulation
G-eHEALS: eHealth Literacy Scale, German version
i2b2: Integrating Biology and the Bedside
ISCED: International Standard Classification of Education
MeSH: Medical Subject Headings
RoBERTa: Robustly Optimized Bidirectional Encoder Representations from Transformers Pretraining Approach
SERP: search engine results page
SNOMED-CT: Systematized Nomenclature of Medicine–Clinical Terms
