# Supplementary material for: Evaluating the Acceptance and Usability of an Independent, Noncommercial Search Engine for Medical Information: Cross-Sectional Questionnaire Study and User Behavior Tracking Analysis
Source: JMIR Hum Factors. 2025 Jan 23;12:e56941. doi: 10.2196/56941 (PMC11803324; doi:10.2196/56941)
Supplement: Multimedia Appendix 1 [file humanfactors_v12i1e56941_app1.pdf]

Search Engine

Search Equation

AppSearch uses Lucene under the hood, which by default relies on the ranking function Okapi BM25 [1] based on the probabilistic relevance framework [2].

Okapi BM25 computes a score of a document D given a query Q containing multiple keywords q_i with the formula

$$\text{score}(D,Q) = \sum_{i=1}^{n} \text{IDF}(q_i) \cdot \frac{f(q_i,D)\cdot(k_1+1)}{f(q_i,D)+k_1\cdot\left(1-b+b\cdot\frac{len(D)}{\text{avgdl}}\right)},$$

where $len(D)$ is the length of the document D and avgdl is the text collection's average document length, both as word counts. k_1 and b are parameters which usually are set to $k_1 \in [1.2, 2.0]$ and $b = 0.75$ [3]. IDF is the inverse document frequency of query term qi which is usually computed as

$$\text{IDF}(q_i) = \log\frac{N - n_i + 0.5}{n_i + 0.5},$$

where N is the number of documents of the collection and n_i is the number of documents the collection containing q_i. (Cf. the elastic search blog [4] for details).

Additionally, AppSearch offered the possibility to store our filter categories as a weighting. A relevance tuning was set up via the user interface, in which weights were set. This has a direct effect on the parameterization of the underlying search equation which we mathematically expressed as

$$\text{score}^*(D^*,Q) = \sum_{f\in F} w(f)\cdot\text{score}(v(D^*,f),Q) + \sum_{c\in C} w(c)\cdot v(D^*,c)$$

where $\text{score}(v(D^*,f),Q)$ refers to the function introduced in Methods and returns the Okapi BM25 score of the specific field value of field f of document D^* which is determined by $v(D,x)$ with $x \in C \cup F$. As parameters we used ElasticSearch's defaults, i.e. $b = 0.75$ and $k_1 = 1.2$ [5]. The set C contains all labels for the constraints and F contains all text field names. Specifically, we defined them as follows:

$$F = \{\text{title,content,url}\}$$
$$C = \{\text{recency,user-friendlyness,comprehensibility,trustworthyness}\}$$

Finally, to weight the different values of the scores of different fields and the fields themselves, the function $w : F \cup C \to \mathbb{R}$ was defined as

$$w(x) = \begin{cases} 8.5, & \text{if } x = \text{content} \\ 7.5, & \text{if } x = \text{title} \\ 2.0, & \text{if } x = \text{url} \\ 5.0, & \text{if } x \in C \end{cases}.$$

These weights were estimated by manually testing various parameters for a limited number of randomly selected medical search terms.

Filter Estimation

The search had 4 filter categories: trustworthiness, recency, user-friendliness and comprehensibility. The scores of these filter categories for a certain domain was computed based on 6 urls, one being the main page and the others randomly selected sub pages. Therefore, we defined a domain as

$$\hat{\mathrm{D}} = \{\mathrm{url}_0^{\hat{\mathrm{D}}}, \mathrm{url}_1^{\hat{\mathrm{D}}}, \mathrm{url}_2^{\hat{\mathrm{D}}}, \mathrm{url}_3^{\hat{\mathrm{D}}}, \mathrm{url}_4^{\hat{\mathrm{D}}}, \mathrm{url}_5^{\hat{\mathrm{D}}}\}$$

For each domain and their sub pages the 74 criteria were estimated, based on which a score for each filter category was computed. The criteria are numbered from 1 to 74; we define $C = \{1, ..., 74\}$. Further, not for every page type, main page and sub page, all criteria were estimated. Hence, not every criterion was available for every url (see Multimedia Appendix 3). The average score of a url was defined to consider only fields which were not valued "not applicable". We mathematically expressed this as follows:

$$m(S \subseteq C, \mathrm{url}) = \frac{1}{n(S, \mathrm{url})} \sum_{k \in S} v(k, \mathrm{url}) \cdot f(k, \mathrm{url})$$

where

$$n(S, \mathrm{url}) = \sum_{k \in S} v(k, \mathrm{url})$$

and

$$v(x, \mathrm{url}) = \begin{cases} 1, & \text{if } f(x, url) \geq 0 \\ 0, & \text{otherwise} \end{cases}$$

$f : C \times U \to \mathbb{R}$ with U containing all available evaluated urls. This function was informally defined to return a numeric value of the field selected in the survey (see Multimedia Appendix 3 for the mappings).

The category ***trustworthiness*** consisted of three sub scores, namely authority, independence and evidence based. ***Authority*** consisted of 6 different questions reflecting if the information about the authority, i.e. author information, of the content of the respective domain were given:

$$\mathrm{A}(\hat{\mathrm{D}}) = \frac{1}{2} \left(m(\{1, 3\}, \mathrm{url}_0^{\hat{\mathrm{D}}}) + \sum_{i=1}^{n=5} m(\{4, ..., 6\}, \mathrm{url}_i^{\hat{\mathrm{D}}}) \right)$$

Next, ***independence*** is estimated. Here, properties such as funding, conflict of interest and whether advertising content is marked were evaluated. If item 7 of a sub page url is "not applicable" it is substituted by $m(\{8, ..., 14\}, \mathrm{url}_i^{\hat{\mathrm{D}}})$.

Thus, we defined a specific function m' to handle that special case

$$m'(S \subseteq C, \mathrm{url}, \mathrm{url}_0) = \begin{cases} m(S, \mathrm{url}), & \text{if } f(7, url) \geq 0 \\ m(\{8, ..., 14\}, \mathrm{url}_0), & \text{otherwise} \end{cases}$$

with which the independence score finally defined as

$$\mathrm{I}(\hat{\mathrm{D}}) = \frac{1}{2}\left(m(\{8, ..., 14\}, \mathrm{url}_0^{\hat{\mathrm{D}}}) + \sum_{i=1}^{n=5} m'(\{7\}, \mathrm{url}_i^{\hat{\mathrm{D}}}, \mathrm{url}_0^{\hat{\mathrm{D}}})\right).$$

The third and last sub-score of trustworthiness reflected if the content was ***evidence based***. Based on 27 questions including one item each for accuracy and comprehensiveness of the sub-pages, which were evaluated by physicians. This score was computed as

$$\mathrm{E}(\hat{\mathrm{D}}) = \frac{1}{3}\left(\frac{1}{2}\left(m(\{15\}, \mathrm{url}_0^{\hat{\mathrm{D}}}) + \sum_{i=1}^{n=5} m(\{16, ..., 41\}, \mathrm{url}_i^{\hat{\mathrm{D}}})\right) + \overbrace{\sum_{i=1}^{n=5} m(\{40\}, \mathrm{url}_i^{\hat{\mathrm{D}}})}^{accuracy} + \overbrace{\sum_{i=1}^{n=5} m(\{41\}, \mathrm{url}_i^{\hat{\mathrm{D}}})}^{comprehensiveness}\right)$$

The final score of trustworthiness of a document $\hat{\mathrm{D}}$ was simply calculated using the average of the sub scores:

$$\mathrm{trustworthiness}(\hat{\mathrm{D}}) = \frac{1}{3}\left(\mathrm{A}(\hat{\mathrm{D}}) + \mathrm{I}(\hat{\mathrm{D}}) + \mathrm{E}(\hat{\mathrm{D}})\right)$$

Recency reflected how new the content was and was computed based on 2 criteria

$$\mathrm{recency}(\hat{\mathrm{D}}) = \frac{1}{2}\left(m(\{43\}, \mathrm{url}_0^{\hat{\mathrm{D}}}) + \sum_{i=1}^{n=5} m(\{42\}, \mathrm{url}_i^{\hat{\mathrm{D}}})\right)$$

Whether a domain $\hat{\mathrm{D}}$ was ***user-friendly*** was estimated using 8 criteria asking for perceived ease of use, such as the existence and embedding of visualizations. It is mathematically defined by

$$\text{user-friendliness}(\hat{\mathrm{D}}) = \frac{1}{2}\left(m(\{44, ..., 49\}, \mathrm{url}_0^{\hat{\mathrm{D}}}) + \sum_{i=1}^{n=5} m(\{50, ..., 53\}, \mathrm{url}_i^{\hat{\mathrm{D}}})\right)$$

The ***comprehensibility*** of the domain $\hat{\mathrm{D}}$ was estimated by inspecting language properties. It was computed by evaluating 20 criteria solely on the sub-pages, which lead to a rather short equation:

$$\mathrm{comprehensibility}(\hat{\mathrm{D}}) = \sum_{i=1}^{n=5} m(\{54, ..., 74\}, \mathrm{url}_i^{\hat{\mathrm{D}}})$$

Finally, after estimating the criteria for each domain, four quantiles were computed, reflecting the filter settings unimportant, rather unimportant, important and very important which were selectable for each filter category. These values were used as thresholds in order to filter documents of the index, which do not fit into the selected filter value.

Literature

1. Robertson SE, Walker S, Jones S, Beaulieu MM, Gatford M. Okapi at TREC-3. In: Proceedings of the 3rd Text REtrieval Conference. 1994. Presented at: TREC-3; November 2-4, 1994; Gaithersburg, MD.
2. Robertson S, Zaragoza H. The probabilistic relevance framework: BM25 and beyond. Found Trends Inf Retr 2009; 3(4):333-89. doi: 10.1561/1500000019
3. Manning CD, Raghavan P, Schütze H. Introduction to Information Retrieval. Cambridge, UK: Cambridge University Press; 2008.
4. Connelly S. Elastic. 2018 Apr 19. Practical BM25 - part 2: the BM25 algorithm and its variables [accessed 2021-04-13] https://www.elastic.co/blog/practical-bm25-part-2-the-bm25-algorithm-and-its-variables
5. Connelly S. Elastic. 2018 Apr 19. Practical BM25 - part 3: considerations for picking b and k1 in Elasticsearch [accessed 2021-04-13] https://www.elastic.co/blog/practical-bm25-part-3-considerations-for-picking-b-and-k1-in-elasticsearch
