# Supplementary material for: Evaluating the Acceptance and Usability of an Independent, Noncommercial Search Engine for Medical Information: Cross-Sectional Questionnaire Study and User Behavior Tracking Analysis
Source: JMIR Hum Factors. 2025 Jan 23;12:e56941. doi: 10.2196/56941 (PMC11803324; doi:10.2196/56941)
Supplement: Multimedia Appendix 2 [file humanfactors_v12i1e56941_app2.pdf]

Appendix 2 - Search Engine Development: List of Domains

Evaluating the Acceptance and Usability of an Independent, Noncommercial Search Engine for Medical Information: Cross-Sectional Questionnaire Study and User Behavior Tracking Analysis

aerzteblatt.de	kindergesundheit-info.de
aponet.de	kinderstarkmachen.de
apotheken-umschau.de	krebsgesellschaft.de
awmf.org	krebshilfe.de
baby-und-familie.de	krebsinformationsdienst.de
bundesgesundheitsministerium.de	leitlinien.de
bzfe.de	leitlinienprogramm-onkologie.de
bzga-essstoerungen.de	loveline.de
dasgehirn.info	lungenaerzte-im-netz.de
deutsche-alzheimer.de	msdmanuals.com
diabetes-ratgeber.net	netdoktor.de
drugcom.de	neurologen-und-psychiater-im-netz.org
familienplanung.de	onmeda.de
gda-portal.de	organspende-info.de
gesundheit.de	patienten-information.de
gesundheit.gv.at	patientenberatung.de
gesundheitsinformation.de	pharmawiki.ch
hilfetelefon.de	rki.de
infektionsschutz.de	senioren-ratgeber.de
iqwig.de	weisse-liste.de
jameda.de	wissenwaswirkt.org
kbv.de	zanzu.de
kenn-dein-limit.de	zecken.de
kinderaerzte-im-netz.de	zentrum-der-gesundheit.de
aerzteblatt.de	kindergesundheit-info.de
gesund.bund.de	tala-med.info

Table S1: *List of domains which were crawled and added to the search index of the search engine as available on the search engine in May 2021. The two green domains were added on 12. Jan 21.*
