# Supplementary material for: Evaluating the Acceptance and Usability of an Independent, Noncommercial Search Engine for Medical Information: Cross-Sectional Questionnaire Study and User Behavior Tracking Analysis
Source: JMIR Hum Factors. 2025 Jan 23;12:e56941. doi: 10.2196/56941 (PMC11803324; doi:10.2196/56941)
Supplement: Multimedia Appendix 4 [file humanfactors_v12i1e56941_app4.pdf]

Appendix 4 - Questionnaire Study: Questionnaire and Detailed Results
Evaluating the Acceptance and Usability of an Independent, Noncommercial Search Engine for Medical Information: Cross-Sectional Questionnaire Study and User Behavior Tracking Analysis

Questionnaire

Full Questionnaire

Page	Content / Questions	Response Options
1	Beginn Umfrage	
	Sehr geehrte Teilnehmerin, sehr geehrter Teilnehmer,<br><br>vielen Dank, dass Sie uns bei der Evaluation der GAP-medinfo Suchmaschine unterstützen.<br><br>In dieser Umfrage wollen wir mehr darüber erfahren, wie Sie mit der Nutzung der Suchmaschine zurechtgekommen sind und wo Sie Verbesserungsbedarf sehen. Es gibt keine richtigen und falschen Antworten, allein Ihr persönlicher Eindruck zählt.<br>Die Umfrage wird ca. 5 Minuten dauern und mit etwas Glück können Sie einen von 25 Büchergutscheinen bei Thalia im Wert von 20€ gewinnen.<br><br>Bevor Sie fortfahren, sollten Sie schon ein wenig mit der Suchmaschine vertraut sein. Wir empfehlen, **ein bis zwei Suchanfragen durchzuführen und sich mit der Trefferliste und den Funktionen vertraut zu machen.**<br><br>Sollten Sie Rückfragen oder sonstige Anmerkungen haben, melden Sie sich gerne per Mail unter *XXX*<br><br>Herzlichen Dank für Ihre Unterstützung,<br>Ihr GAP-medinfo Team<br><br>***Vorab eine Bemerkung zum Datenschutz, eine ausführlichere Beschreibung zur Studie und Ihre Einwilligung zum Datenschutz finden Sie auf der nächsten Seite.***<br>*Dies ist eine anonyme und freiwillige Umfrage.*<br>*In den Umfrageantworten werden keine persönlichen Informationen über Sie gespeichert.*<br>*Am Ende der Umfrage können Sie über einen neuen Link Ihre Emailadresse eingeben, um an unserem Gewinnspiel (einer von 25 Thalia-Büchergutscheinen im Wert von 20€) teilzunehmen. Ihre Emailadresse wird* ***nicht*** *zusammen mit Ihren Angaben in den Fragen abgespeichert. Sie wird getrennt aufbewahrt und direkt nach Durchführung des Gewinnspiels wieder gelöscht.*<br>*Weitere Hinweise zum Datenschutz entnehmen Sie bitte dem Impressum auf der Website der Suchmaschine.*	
2	Willkommen	
	**Bevor Sie beginnen, lesen Sie bitte die folgenden Informationen und bestätigen Sie Ihre Einwilligung.**	
	**Informationen zur Befragung**<br>**Hintergrund**<br>Ein Ziel des Projekts „Gut informierte Kommunikation zwischen Arzt und Patient (GAP)“ ist es, BürgerInnen und PatientInnen verständliche Gesundheitsinformationen zur Verfügung zu stellen. Dazu wurde eine Suchmaschine entwickelt, die der Nutzerin/ dem Nutzer helfen soll, die Qualität von Suchergebnissen mit Hilfe der vier Kategorien Verständlichkeit, Vertrauenswürdigkeit, Nutzerfreundlichkeit und Verständlichkeit selbst einzuschätzen.<br>Um die Akzeptanz und die Nutzbarkeit der Suchmaschine zu bewerten, werden Personen, die die Suchmaschine genutzt haben, zu Ihrer Nutzung der Suchmaschine und zu Ihrem Eindruck von der Suchmaschine befragt. Die	

	Befragung wird vom Institut für Allgemeinmedizin des Universitätsklinikums Freiburg betreut und dauert nur wenige Minuten.<br>**Teilnahmeende/Widerruf**<br>Die Teilnahme an der Befragung ist freiwillig und anonym. Im Falle einer Nichtteilnahme entstehen Ihnen keinerlei Nachteile. Wenn Sie daran teilnehmen, können Sie Ihre Teilnahme jederzeit ohne Angabe von Gründen abbrechen. Schließen Sie dazu einfach den Browser. Die Suchmaschine kann auch unabhängig von der Umfrage weiter genutzt werden.<br>**Einwilligung Datenschutz**<br>Die gesetzlichen Bestimmungen des Datenschutzes werden streng eingehalten. In der gesamten Befragung werden keine Merkmale erfragt, aufgrund derer Sie identifiziert werden könnten. Es werden lediglich wenige allgemeine Merkmale zu Ihrer Person erfragt. Dies sind: Alter, Geschlecht, Schulabschluss, beruflicher Kontext und Zugehörigkeit zu Fachgesellschaften. Zur Wahrung der Anonymität bitten wir Sie, auch bei Einträgen in Freitextfelder keine Angaben zu äußern, die Sie als Person eindeutig erkennen lassen. Ihre anonymen Angaben aus der Befragung können ganz oder in Ausschnitten in Forschungsberichten, Fortbildungen oder weiteren Veröffentlichungen verwendet werden. Eine rückwirkende Löschung Ihrer Angaben ist nicht möglich.<br>**Datenaustausch**<br>Ihre Angaben in den Fragebogen werden nur in den am Projekt GAP beteiligten datenauswertenden Instituten ausgetauscht. Dies sind das Universitätsklinikum Freiburg (vertreten durch Cochrane Deutschland und den Lehrbereich Allgemeinmedizin) und die Universität Freiburg (vertreten durch Medical Data Science). Da Ihre Angaben anonym sind, können auch diese auswertenden Institute nicht auf Ihre Person rückschließen.<br>**Rückfragen**<br>Falls Sie Rückfragen zu Ablauf und Inhalt der Befragung haben, können Sie sich an folgende Ansprechpartnerin wenden:<br>XXX	
	Einverständniserklärung	
	1. Hiermit bestätige ich, dass ich über den Inhalt und die Zielsetzung der Befragung zur Suchmaschine des Projektes „Gut informierte Kommunikation zwischen Arzt und Patient (GAP)“ informiert worden bin. Ich hatte ausreichend Bedenkzeit und alle meine Fragen wurden zufriedenstellend beantwortet.<br>2. Ich wurde darüber informiert, dass ich das Recht habe, die Teilnahme an der Befragung jederzeit ohne Angabe von Gründen abzubrechen. Eine rückwirkende Löschung meiner Forschungsdaten (d.h. Angaben in den Fragebögen) ist nicht möglich. Mir entstehen weder aus der Teilnahme noch aus der Nichtteilnahme Nachteile.<br>3. Ich erkläre mich einverstanden, dass meine Daten wie in den Informationen zur Befragung beschrieben ausgetauscht und ausgewertet werden.<br>4. Die Verantwortlichen tragen Sorge dafür, dass alle erhobenen Daten streng vertraulich behandelt und ausschließlich im Rahmen der Zweckbestimmung dieser Studie verwendet werden.	(Checkbox)<br>Unter den hier aufgeführten und in den obigen Informationen genannten Bedingungen erkläre ich mich bereit, an der Befragung teilzunehmen
3.1	keine Einwilligung	

	**Sie haben keine Einwilligung zur Befragung erteilt. Ohne Ihre Einwilligung ist eine Teilnahme leider nicht möglich.**<br>Falls Sie sich verklickt haben, klicken Sie auf "Zurück" und korrigieren Sie Ihre Angabe. Um Ihre Auswahl (keine Einwilligung und somit keine Teilnahme an der Umfrage) zu bestätigen, klicken Sie auf "weiter" und beendet hiermit die Umfrage.	
3.2	Filter-keine Einwilligung	
	**Herzlichen Dank für Ihr Interesse!**<br>Es wurden keine Daten übermittelt, Sie können das Browserfenster jetzt schließen.<br>Bei Rückfragen zur Befragung können Sie sich gerne an folgenden Ansprechpartner wenden: XXX	
4	Angaben zur Person anonym	
	**Wie alt sind Sie?**<br>*Bitte geben Sie die Altersgruppe an, zu der Sie zählen.*	jünger als 18 Jahre / 18 - 29 Jahre / 30 - 39 Jahre / 40 - 49 Jahre / 50 - 59 Jahre / 60- 69 Jahre / 70- 79 Jahre / älter als 80 Jahre
	**Bitte geben Sie Ihr Geschlecht an:**	divers / weiblich / männlich
	**Was ist Ihr höchster Bildungsabschluss?**	kein Abschluss / Hauptschulabschluss / Realschulabschluss / Fachabitur/ Abitur / Universitäts-/ Hochschulabschluss / Promotion
	**Arbeiten Sie im medizinischen Bereich?**	ich arbeite als Ärzt*in / ich arbeite in einem anderen Heilberuf / ich bin in keinem Heilberuf tätig
	**Gehören Sie einer "spezifischen Gruppe" an, die Sie auf die Umfrage aufmerksam gemacht hat?**<br>*Bitte geben Sie hier an, welcher "Gruppe" Sie angehören.*	keine der genannten Gruppen / "Gesundes Kinzigtal" / Deutsche Gesellschaft für Allgemeinmedizin und Familienmedizin e.V. (DEGAM) / Deutsches Netzwerk Versorgungsforschung e.V. (DNVF) / Deutsche Gesellschaft für Innere Medizin e.V. (DGIM) / Deutsche Gesellschaft für Orthopädie und Unfallchirurgie (DGOU) / Deutsche Gesellschaft für Chirurgie (DGCH) / Deutsche Gesellschaft für Rehabilitationswissenschaften e.V. (DGRW) / Deutsche Gesellschaft für Arbeitsmedizin und Umweltmedizin (DGAUM) / andere medizinische Fachgesellschaft / WISO-Panel
5	Webaffinität	
	**Wie regelmäßig nutzen Sie das Internet, um sich über Gesundheitsthemen zu informieren?**<br>(Bitte beantworten Sie die Frage, wenn möglich, wie Sie es vor dem aktuellen Coronavirus gemacht haben.)<br>*Bitte wählen Sie eine der folgenden Antworten:*	Täglich / mehrmals pro Woche / mehrmals pro Monat / seltener / nie
	**Wie würden Sie Ihre Erfahrungen und Einstellungen zur Internetnutzung für Gesundheitsinformationen einschätzen?**<br>*Bitte geben Sie für die beiden Aussagen an, inwieweit diese auf Sie zutreffen.*	
	Ich kann im Internet zuverlässige von fragwürdigen Informationen unterscheiden.	trifft überhaupt nicht zu / trifft eher nicht zu / trifft teilweise zu / trifft eher zu / trifft voll und ganz zu

	Wenn ich gesundheitsbezogene Entscheidungen für mich oder andere auf Basis von Informationen aus dem Internet treffe, fühle ich mich dabei sicher.	
6	Usability	
	**Nachfolgend finden Sie einige Aussagen über die Suchmaschine.** *Bitte geben Sie jeweils an, inwiefern Sie der Aussage zustimmen.*	
	Ich kann mir vorstellen, dass die meisten Personen den Umgang mit der Suchmaschine sehr schnell lernen.	trifft überhaupt nicht zu / trifft eher nicht zu / trifft teilweise zu / trifft eher zu / trifft voll und ganz zu
	Das Design unterstützt ein schnelles Scannen der Suchergebnisseiten.	
	Ich finde die Elemente der Suchergebnisse sind übersichtlich angeordnet.	
	Die Einstellungen der Funktionen der Suchmaschine sind für mich nicht verständlich.	
	Dass es hier keinerlei Werbung gibt, gefällt mir.	
	Die Suchfunktionen sind genauso, wie ich es erwartet habe.	
	Die Suchmaschine geht sorgsam mit meinen persönlichen Daten um.	
	Die Suchmaschine liefert Suchergebnisse, die nicht durch wirtschaftliche Interessen beeinflusst werden.	
	Die Suchmaschine liefert schnell die Information, die man braucht.	
	Die Suchmaschine bietet sinnvolle Optionen, die bisherige Suchmaschinen so nicht beinhalten.	
7	Innovationsaspekte	
	**Die soeben von Ihnen getestete Suchmaschine legt im Gegensatz zu großen kommerziellen Suchmaschinen besonderen Wert darauf, dass Ihre Suchanfragen anonym bleiben und NICHT zur Erstellung von Profilen genutzt werden und dass die Reihenfolge der Suchergebnisse NICHT durch Sponsoren beeinflusst wird.** Uns würde interessieren, ***wie wichtig Ihnen*** diese beiden Aspekte bei der Nutzung einer Suchmaschine sind.	völlig unwichtig / eher unwichtig / weder unwichtig noch wichtig / eher wichtig / sehr wichtig
8	Fragen zu Kategorien	
	**Im Folgenden finden Sie Fragen zu den in unserer Suchmaschine spezifischen Elementen.**	

Appendix 4 - Questionnaire Study: Questionnaire and Detailed Results
Evaluating the Acceptance and Usability of an Independent, Noncommercial Search Engine for Medical Information: Cross-Sectional Questionnaire Study and User Behavior Tracking Analysis

<table>
<tr><td rowspan="5"></td><td colspan="2">Bitte geben Sie an, inwieweit die jeweilige Aussage zur Suchmaschine auf Sie zutrifft.</td></tr>
<tr><td>Der Filter "vertrauenswürdig" hilft mir tatsächlich, vertrauenswürdige Ergebnisse zu finden.</td><td rowspan="4">stimme ich überhaupt nicht zu / stimme ich eher nicht zu / stimme ich teilweise zu / stimme ich eher zu / stimme ich voll und ganz zu / Ich habe die Funktion nicht benutzt, ich kann diese Frage nicht bewerten.</td></tr>
<tr><td>Der Filter "aktuell" hilft mir tatsächlich, aktuelle Ergebnisse zu finden.</td></tr>
<tr><td>Der Filter "verständlich" hilft mir tatsächlich, verständliche Ergebnisse zu finden.</td></tr>
<tr><td>Der Filter "nutzerfreundlich" hilft mir tatsächlich, nutzerfreundliche Ergebnisse zu finden.</td></tr>
<tr><td rowspan="5">9</td><td colspan="2">Wiedernutzung?</td></tr>
<tr><td colspan="2">Jetzt, wo Sie die Suchmaschine getestet und bewertet haben, was denken Sie zu folgender Aussage:</td></tr>
<tr><td>Ich denke, dass ich die Suchmaschine häufig benutzen würde.</td><td rowspan="2">stimme überhaupt nicht zu / stimme eher nicht zu / stimme teilweise zu / stimme eher zu / stimme voll und ganz zu</td></tr>
<tr><td>Ich denke, dass ich die Suchmaschine weiterempfehlen werde.</td></tr>
<tr><td>Zum Schluss haben Sie noch die Möglichkeit, eigene Anmerkungen oder Verbesserungsvorschläge einzubringen:
Vielleicht möchten Sie noch was zur Suchmaschine "loswerden", oder Sie haben bemerkt, dass etwas Wichtiges fehlt, oder haben noch einen wichtigen Gedanken dazu - Hier ist Platz diesen auszuformulieren:</td><td>(Textfeld)</td></tr>
<tr><td rowspan="2">10</td><td colspan="2">Endseite</td></tr>
<tr><td colspan="2">Vielen Dank, dass Sie sich die Zeit genommen haben, an der Umfrage teilzunehmen!
Durch Ihre Hilfe können wir die Suchmaschine weiterentwickeln und an die Bedürfnisse der Nutzerinnen und Nutzer anpassen.

Wenn Sie an unserem Gewinnspiel teilnehmen wollen, klicken Sie bitte auf folgenden Link: Teilnahme am Gewinnspiel
Hier können Sie dann Ihre Emailadresse eingeben.

Beim Gewinnspiel können Sie einen von 25 Büchergutscheinen bei Thalia im Wert von 20€ gewinnen.
Ihre Email-Adresse gilt als Grundlage zur Verlosung, so dass wir Sie darüber im Falle eines Gewinnes kontaktieren können.
Die Email-Adressen werden unabhängig von Ihren Antworten gespeichert, sodass die Anonymität Ihrer Antworten gewahrt bleibt. Unmittelbar nach der Verlosung werden alle Email-Adressen gelöscht.

Sollten Sie nicht teilnehmen wollen, ist die Umfrage hiermit beendet.</td></tr>
</table>

Table S1: *Full Questionnaire; original survey in German.*

Appendix 4 - Questionnaire Study: Questionnaire and Detailed Results
Evaluating the Acceptance and Usability of an Independent, Noncommercial Search Engine for Medical Information: Cross-Sectional Questionnaire Study and User Behavior Tracking Analysis

Content of the Questionnaire

Content		Utilized scales	Response scales	Number of items
**Participants**				
Sociodemographic information		-		4
Frequency of internet use for health information		-	daily (1) – several times a week (2) – several times a month(3) – less frequently (4) – never (5)	1
self-perceived digital health literacy				
	Searching for information	Individual items from G-eHEALS (Söllner et al. 2014; 10 items, 2 subscales): one item from each subscale	Likert scale: strongly disagree (1) – disagree (2) – partially agree (3) – agree (4) – strongly agree (5)	2
	Assessing information			
**Evaluation of the search engine**				
Usability				
	Topics: learnability, efficient scannability of SERP, clarity of SERP, comprehensibility of functions, absence of advertising, expectations, handling of personal data, absence of commercial bias, fast access to relevant information, innovative approach	Items from Magin et al. 2015, Quirmbach 2012 (adapted version of the system usability scale (SUS)), and own items	Likert scale: strongly disagree (1) – disagree (2) – partially agree (3) – agree (4) – strongly agree (5)	10
Acceptance				
	Potential re-use	From adapted version of the SUS (Quirmbach 2012)	Likert scale: strongly disagree (1) – disagree (2) – partially agree (3) – agree (4) – strongly agree (5)	2
	Recommendation to others	-		
Innovative aspects of the search engine (importance of anonymous searches)		-	Likert scale: very unimportant (1) – unimportant (2) – neither unimportant or important (3) – important (4) – very important (5)	1
Helpfulness of filters				
	For each filter (trustworthiness, recency, user-friendliness and comprehensibility)	-	Likert scale: strongly disagree (1) – disagree (2) – partially agree (3) – agree (4) – strongly agree (5)	4
Further comments or suggestions for improvements			Open-ended question with text box	1
				Total: 25

Table S2*: Detailed content of the questionnaire with utilized scales*

Detailed Results

Participants

		n	%	Mean	SD
**Age**				4,78	1,41
	< 18 (1)	0	0		
	18-29 (2)	47	5.9		
	30-39 (3)	128	16.0		
	40-49 (4)	141	17.6		
	50-59 (5)	215	26.8		
	60-69 (6)	189	23.6		
	70-79 (7)	75	9.4		
	>80 (8)	7	0.9		
**Gender**					
	divers	4	0.5		
	female	432	53.9		
	male	366	45.6		
**Occupation**					
	medical doctor	8	1.0		
	other medical profession	68	8.5		
	no medical profession	726	90.5		
**Highest level of educational attainment**				4.13	1.08
	no formal secondary education (“*kein Abschluss*”, ISCED-2011 0-1) (1)	3	0.4		
	lower secondary education (“*Hauptschulabschluss*”, ISCED-2011 level 2) (2)	58	7.2		
	lower secondary education (“*Realschulabschluss*”, ISCED-2011 level 2) (3)	185	23.1		
	upper secondary education ("*Fachabitur oder Abitur*”, ISCED-2011 level 3-4) (4)	184	22.9		
	tertiary education: bachelor's or master’s degree or equivalent (ISCED-2011 level 6-7) (5)	330	41.1		
	doctorate degree (ISCED-2011 level 8) (6)	42	5.2		
**frequency of internet use for health information**				2.99	0.97
	daily (1)	74	9.2		
	several times a week (2)	148	18.5		
	several times a month (3)	302	37.7		
	less frequently (4)	266	33.2		
	never (5)	12	1.5		
**self-perceived digital health literacy:** searching for information – sense of security when making decisions based on information on the internet				3.19	0.90
	strongly disagree (1)	31	3.9		
	disagree (2)	121	15.1		
	partially agree (3)	359	44.8		

	agree (4)	245	30.5		
	strongly agree (5)	46	5.7		
**self-perceived digital health literacy:** assessing information – ability to assess reliability of websites				3.59	0.86
	strongly disagree (1)	16	2.0		
	disagree (2)	55	6.9		
	partially agree (3)	270	33.7		
	agree (4)	363	45.3		
	strongly agree (5)	98	12.2		

Table S3: *Participants; n = 802*

Evaluation of the Search Engine

Acceptance

	strongly disagree (1): n (%)	disagree (2): n (%)	partially disagree (3): n (%)	agree (4): n (%)	strongly agree (5): n (%)	**Mean**	**SD**	**Cronbach α**
**Acceptance**						3.63	0.98	0.90
Item 1: potential re-use	23 (2.9)	101 (12.6)	213 (26.6)	319 (39.8)	146 (18.2)	3.58	1.02	
Item 2: recommendation to others	28 (3.5)	82 (10.2)	185 (23.1)	332 (41.4)	175 (21.8)	3.68	1.03	

Table S4: *Acceptance; n = 802*

Usability

	strongly disagree (1): n (%)	disagree (2): n (%)	partially disagree (3): n (%)	agree (4): n (%)	strongly agree (5): n (%)	**Mean**	**SD**	**Cronbach α**
**Usability**						3.76	0.61	0.83
Item 1: quick to learn	10 (1.2)	33 (4.1)	123 (15.3)	392 (48.9)	244 (30.4)	4.03	0.86	
Item 2: efficient scannability of SERP	23 (2.9)	91 (11.3)	193 (24.1)	351 (43.8)	144 (18.0)	3.63	1.00	
Item 3: clarity of SERP	19 (2.4)	76 (9.5)	179 (22.3)	338 (42.1)	190 (23.7)	3.75	1.00	
Item 4: functions NOT comprehensible	259 (32.3)	236 (29.4)	125 (15.6)	115 (14.3)	67 (8.4)	2.37[x]	1.29	
Item 5: appreciation for the absence of advertising	5 (0.6)	6 (0.7)	44 (5.5)	149 (18.6)	598 (74.6)	4.66	0.66	
Item 6: functionality meets expectations	33 (4.1)	113 (14.1)	230 (28.7)	313 (39.0)	113 (14.1)	3.45	1.03	
Item 7: careful handling of personal data	18 (2.2)	44 (5.5)	303 (37.8)	324 (40.4)	113 (14.1)	3.59	0.88	
Item 8: no commercial bias	25 (3.1)	68 (8.5)	268 (33.4)	320 (39.9)	121 (15.1)	3.55	0.95	
Item 9: fast access to relevant information	20 (2.5)	68 (8.5)	182 (22.7)	341 (42.5)	191 (23.8)	3.77	0.99	

Item 10: innovative approach	30 (3.7)	81 (10.1)	241 (30.0)	291 (36.3)	159 (19.8)	3.58	1.03	

Table S5: *Usability; n = 802; ˣ item 4 (comprehensibility of functions) was a negated question and therefore adjusted for calculating the scale values (see Figure 4)*

Importance of Anonymous Searches

	very unimportant (1): n (%)	unimportant (2): n (%)	neither unimportant or important (3): n (%)	important (4): n (%)	very important (5): n (%)	**Mean**	**SD**
**Innovative aspects** of the search engine (anonymous searches)	3 (0.4)	24 (3.0)	42 (5.2)	231 (28.8)	502 (62.6)	4.50	0.76

Table S6: *Importance of anonymous searches; n = 802*

Helpfulness of Filters

	n (% of 802)	strongly disagree (1): n (%)	disagree (2): n (%)	partially agree (3): n (%)	agree (4): n (%)	strongly agree (5): n (%)	**Mean**	**SD**
**Helpfulness of filters** for each filter								
Item 1: trustworthiness	599 (74.7)	12 (2.0)	20 (3.3)	93 (15.5)	241 (40.2)	233 (38.9)	4.11	0.92
Item 2: recency	622 (77.6)	5 (0.8)	17 (2.7)	79 (12.7)	235 (37.8)	286 (46.0)	4.25	0.84
Item 3: comprehensibility	589 (73.4)	9 (1.5)	27 (4.6)	106 (18.0)	230 (39.0)	217 (36.8)	4.05	0.93
Item 4: user-friendliness	561 (70.0)	9 (1.6)	34 (6.1)	114 (20.3)	243 (43.3)	161 (28.7)	3.91	0.93

Table S7: *Helpfulness of filters; n=total number of respondents who indicated that they used the filter functions listed for each item (all other participants answered: "I did not use this function, I cannot evaluate this question")*
