# Supplementary material for: Evaluating the Acceptance and Usability of an Independent, Noncommercial Search Engine for Medical Information: Cross-Sectional Questionnaire Study and User Behavior Tracking Analysis
Source: JMIR Hum Factors. 2025 Jan 23;12:e56941. doi: 10.2196/56941 (PMC11803324; doi:10.2196/56941)
Supplement: Multimedia Appendix 5 [file humanfactors_v12i1e56941_app5.pdf]

Appendix 5 - User-behavior Tracking Details

Evaluating the Acceptance and Usability of an Independent, Noncommercial Search Engine for Medical Information: Cross-Sectional Questionnaire Study and User Behavior Tracking Analysis

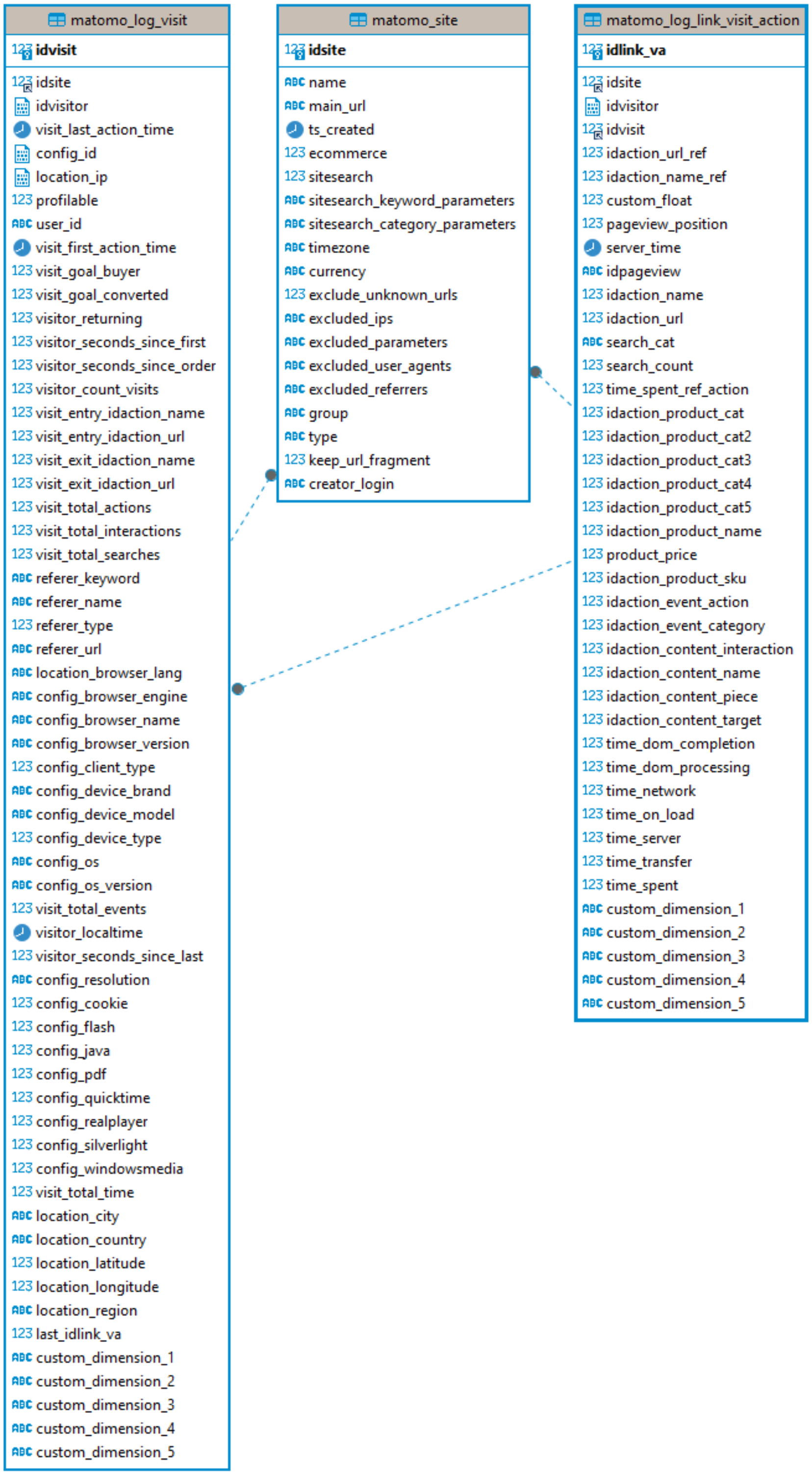

Figure S1: *Entity relationship diagram of Matomo's database. For each tracked website (matomo_site) Matomo saves the visits (matomo_log_visit saves) with their actions (matomo_log_link_visit_action).*

Appendix 5 - User-behavior Tracking Details

Evaluating the Acceptance and Usability of an Independent, Noncommercial Search Engine for Medical Information: Cross-Sectional Questionnaire Study and User Behavior Tracking Analysis

corona	124
rückenschmerzen	38
husten	36
diabetes	24
depression	24
covid-19	22
kopfschmerzen	22
rückenschmerz	20
rücken	19
migräne	19
brustkrebs	18
asthma	15
krebs	14
covid	12
cholesterin	12
rheuma	12
schilddrüse	11
corona impfung	11
arthrose	11
covid 19	11
knieschmerzen	10
heuschnupfen	10
schlaganfall	10
umfrage	9
darmkrebs	9

Table S1: Top 25 search terms with frequency of the 3090 unique search terms.
